# Bleeding Complications in Patients Undergoing Percutaneous Coronary Intervention

**DOI:** 10.31083/j.rcm2308286

**Published:** 2022-08-15

**Authors:** Mattia Galli, Renzo Laborante, Felicita Andreotti, Rocco Vergallo, Rocco Antonio Montone, Antonio Iaconelli, Carlo Trani, Francesco Burzotta, Filippo Crea, Domenico D’Amario

**Affiliations:** ^1^Department of Cardiovascular and Pulmonary Sciences, Catholic University of the Sacred Heart, 00168 Rome, Italy; ^2^Department of Cardiology, Maria Cecilia Hospital, GVM Care & Research, 48033 Cotignola, Italy; ^3^Department of Cardiovascular Medicine, Fondazione Policlinico Universitario A. Gemelli IRCCS , 00168 Rome, Italy

**Keywords:** percutaneous coronary interventions, bleeding, complications, antithrombotic therapy

## Abstract

Percutaneous coronary intervention (PCI) is considered a relatively safe procedure associated with low rates of complications, but is inevitably associated with short and mid-to-long term increased bleeding risk. Besides the short term risk associated with the arterial access to perform PCI, enhanced bleeding risk persists for several months, given the need for antithrombotic therapy to prevent procedure-related thrombotic complications as well as ischemic recurrences. Bleeding is a powerful harbinger of adverse outcomes. This awareness has fuelled intense research on bleeding reduction strategies, including new PCI devices and techniques as well as new medications and antithrombotic regimens. We here review the mechanisms and prevalence of bleeding in PCI patients, discuss the available evidence from a practical point of view, and explore future perspectives on how to treat and prevent bleeding complications in these patients.

## 1. Introduction

Since the first coronary angioplasty performed by Andreas Grüntzig in 1977, there has been significant progress in the field of percutaneous coronary intervention (PCI), which currently represents a cornerstone in the management of ischemic heart disease (IHD) [1]. PCI, however, requires an arterial vascular access and adjunctive antithrombotic therapy, such as intraprocedural parenteral anticoagulation, as well as mid-to-long-term dual antiplatelet therapy (DAPT), consisting in the association of aspirin plus a ${\mathrm{P2Y}}_{12}$ inhibitor [2]. The former is mainly needed to prevent acute thrombosis caused by interactions of device surfaces with the bloodstream, while the latter plays a key role in preventing local ischemic events, such as stent thrombosis (ST), and myocardial infarction (MI) [2]. These treatments come at the cost of increased bleeding. According to the SWEADHEART registry, in the period from 1995 to 2018, i.e., the years in which PCI has been increasingly implemented, the incidence of in-hospital and out-of-hospital bleeding in patients with acute coronary syndrome (ACS) has doubled (2.9% vs. 6.3%), albeit with a parallel overall significant survival benefit (24.4% vs. 14.6%) [3]. Robust evidence shows that major bleeding is independently associated with adverse prognosis, including increased mortality [4]. Even minor bleeding carries important prognostic implications, leading to abrupt discontinuation of antiplatelet therapy resulting in higher ischemic events [5, 6]. To this extent, there has been a growing interest in the development of bleeding reduction strategies aiming at reducing bleeds without any trade-off in thrombotic complications after PCI over the past two decades [5]. Nowadays, the adoption of strategies to reduce bleeding and of antithrombotic regimens with a more favorable safety to efficacy balance, tailored to individual patient characteristics, has become an essential step to improve patient prognosis after PCI. We here review the mechanisms and prevalence of bleeding in PCI patients, and discuss the available evidence on its treatment and prevention from a practical point of view.

## 2. Bleeding Definitions 

**Fig. 1.**
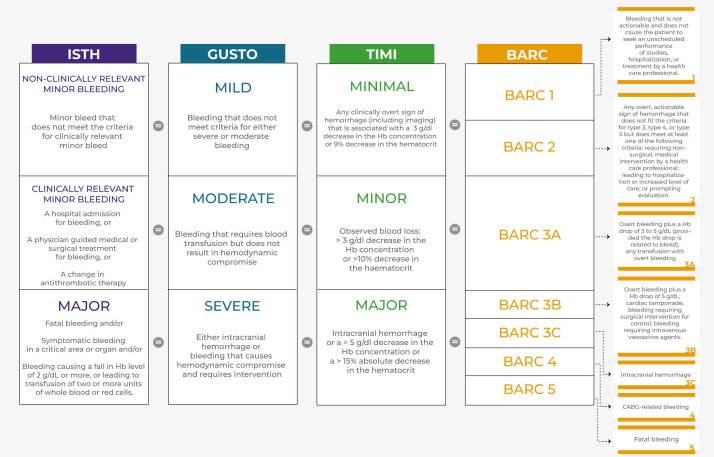
**Comparison of the most used bleeding classifications**. ISTH classifies bleeding according to clinical relevance. TIMI, GUSTO and BARC represent the most used bleeding classifications. ISTH, TIMI and GUSTO are divided into three levels of severity, while BARC is divided into five, numerically graded, levels. Prognostically, a bleed classified as “mild” by GUSTO corresponds to a “minimal” bleed by TIMI and to a “1” or “2” bleed by BARC. A bleed classified as “moderate” according to GUSTO, corresponds to a “minor” bleed by TIMI and to a “3a” bleed according to BARC. Finally, a bleed classified as “severe” according to GUSTO corresponds to a “major” bleed according to TIMI and to a “3b”, “3c”, “4” or “5” bleed according to BARC. Abbreviations. ISTH, International Society on Thrombosis and Haemostasis; BARC, Bleeding Academic Research Consortium; GUSTO, Global Use of Strategies to Open Occluded Coronary Arteries; TIMI, The Thrombolysis in Myocardial Infarction.

The first challenge when dealing with bleeding among PCI patients stems from the fact that bleeding is a complex clinical phenomenon, which is hard to enclose under an univocal classification, given its broad range of severities, sites, and hemodynamic consequences. Several bleeding definitions have been proposed over time, generating some confusion and hindering comparisons of incidence and prognostic relevance of bleeds across different studies [7, 8] (Fig. 1).

The first widely used definitions were the GUSTO (Global Use of Strategies to Open Occluded Arteries) and the TIMI (Thrombolysis in Myocardial Infarction), developed in patients with ST-elevation myocardial infarction (STEMI) receiving thrombolytic therapy [9, 10]. These definitions were designed to classify relatively severe bleeds, and are suboptimal in capturing less severe events typical of the post-fibrinolytic era [8]. The TIMI classification stratifies events according to coronary artery bypass graft (CABG) or not. It is mainly based on laboratory parameters (fall in hematocrit or hemoglobin), with the limitation of not specifying their timing, which may lead to changeable peaks and nadirs [8, 9].

Converseley, the GUSTO classification is mainly driven by life-threatening (major) bleeds, such as intracranial or hemodynamically unstable, and those requiring transfusion in the absence of hemodynamic instability (moderate bleeds) [8, 10]. Because transfusion criteria may vary according to local clinical practice, adjudication may not be consistent across geographic regions [8].

In 2005, the International Society on Thrombosis and Haemostasis (ISTH) defined bleeding as major, stratified by surgery or not, or minor, stratified by clinical relevance or not [11]. This definition groups heterogeneous events of variable severity, presentation and course, and has been less implemented in recent years [12].

In the attempt to overcome the limitations of previous classifications, several trials have created their own definitions, combining elements from both TIMI and GUSTO definitions and adding new parameters [8]. Notable examples include the CURE, ACUITY, OASIS, STEEPLE and PLATO definitions [8]. The PLATO definition, for example, classifies bleeds as major (life-threatening or not), minor or minimal [8]. PLATO major bleeds include a broader range of events compared to TIMI or GUSTO criteria. Thus, bleeding with a drop in hemoglobin of 3 to 5 g/dL would be defined as major by PLATO and minor by TIMI criteria, while a clinically stable event requiring transfusion would be considered PLATO major and GUSTO moderate. Trial specific definitions present the inherent limitation of hindering the comparison of incidence and of the prognostic impact of bleeding across studies.

More recently, the Bleeding Academic Research Consortium (BARC), uniting academia, professional societies and federal agencies, has focused on clinical (such as health care intervention or harmfulness of bleeding site) and laboratory criteria (such as hematocrit and hemoglobin) to classify bleeds, using ordinal numbers rather than qualitative terms, and showing increasing bleeding severity and mortality with increasing BARC grades [8].

Because the BARC classification captures a larger proportion of clinically significant bleeding than the GUSTO or TIMI scales and provides more precise sub-classifications, it has been extensively adopted over the years, becoming the most used and reliable bleeding definition.

## 3. Incidence of Bleeding After PCI

**Fig. 2.**
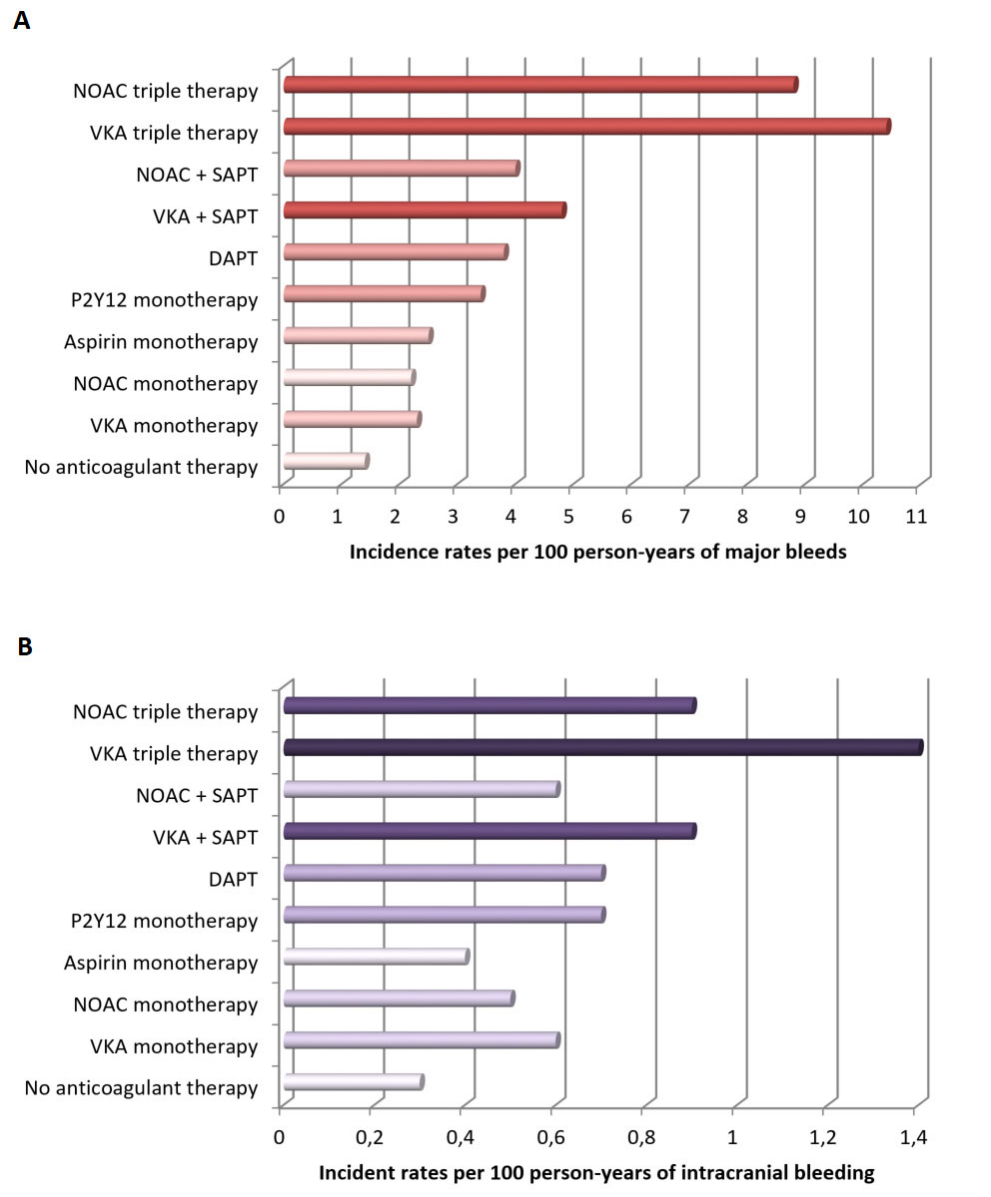
**Bleeding incidence associated with single, dual, and triple therapy (*modified from Nienke van Rein et al*. [14])**. (A) Incidence rates of major bleeding. (B) Incidence rates of intracranial hemorrhage. Abbreviations. DAPT, dual antiplatelet therapy; NOAC, non-vitamin K antagonist oral anticoagulant; VKA, vitamin K antagonist; SAPT, single antiplatelet therapy.

Bleeding is one of the most common complications in patients treated with DAPT or with the combination of antiplatelet agents and oral anticoagulation (OAC), typical of patients with atrial fibrillation (AF) undergoing PCI [5]. The risk of bleeding is proportional to the intensity and duration of antithrombotic treatment [4, 13] (Fig. 2, Ref [14]).

In fact, patients with AF on triple antithrombotic therapy (TAT) experience higher rates of major bleeding compared to patients on dual antithrombotic therapy (DAT, i.e., single antiplatelet therapy plus OAC) or DAPT [14]. In a national Danish cohort of 272,315 patients with AF aged 50 years or older, during a one year follow-up, major bleeding was lowest in patients not treated with any antithrombotic agent and increased with the number of anticoagulants or antiplatelet drugs, with incidence rates between 1.3 and 10.4 per 100 patient-years (Fig. 2) [14]. Importantly, the type of ${\mathrm{P2Y}}_{12}$ inhibitor or OAC used is a major determinant of major and intracranial bleeding. Indeed, incidence rates for major bleeding are lower with non-vitamin K antagonist OAC (NOAC) compared to vitamin K antagonist (VKA), and to clopidogrel compared with prasugrel or ticagrelor [15, 16]. Intracranial hemorrhage is one of the most dreaded complications of antithrombotic therapy, with incidence rates between 0.4 and 1.4 per 100 person-years, being highest among patients treated with TAT including a VKA [17]. Of note, the use of NOACs is associated with a marked reduction of intracranial hemorrhage compared to VKA [18].

As expected, bleeding rates are significantly increased in patients defined as being at high-bleeding risk (HBR). In fact, these patients have a 38% higher risk of major or clinically relevant non-major bleeding and a 71% higher risk of major bleeding compared to their non-HBR counterparts [19]. Therefore, stratification aimed at identifying HBR patients is crucial as discussed below [5].

## 4. Clinical Implications of Bleeding After PCI

Bleeding complications can occur in-hospital or post-discharge. In-hospital, the Cath-PCI registry of >3 million PCI patients in USA between 2004 and 2011 reported, at on propensity-matched cohort analysis, that major bleeding was associated with increased in-hospital mortality (5.2% vs. 1.8%) [20]. Furthermore, both access-site and non-access site bleeding were associated with increased in-hospital mortality (2.7% vs. 1.8% and 8.2% vs. 1.8%, respectively) [21]. Interestingly, non-access site bleeding showed a stronger association with mortality compared to access-site, a finding which is in contrast with the common belief that in-hospital morality due to bleeding is mainly related to access-site bleeding and underlines the need for bleeding reduction strategies early during the hospital stay [5].

Out-of-hospital, data from 8582 patients enrolled in the ADAPT-DES study showed a strong association of post-discharge bleeding vs. none with all-cause mortality (13.0% vs. 3.0%) [22]. Importantly, as compared to post-discharge MI, post-discharge bleeding had an even greater effect on subsequent mortality (hazard ratio HR, 1.92; 95% confidence interval, CI 1.18 to 3.12; *p* = 0.009) [22].

The association between MI, post-discharge bleeding and all-cause mortality was also elegantly assessed by a post-hoc analysis of the TRACER trial that included 12,944 patients with non-ST Elevation MI (NSTEMI) [4]. In this study, MI was associated with a greater risk of mortality compared with BARC 2 (relative risk, RR, 3.5; 95% CI 2.08–4.77; *p *< 0.001) and BARC 3a bleeding (RR 2.23; 95% CI 1.36–3.64; *p* = 0.001). Nevertheless, the risk of mortality associated with MI was similar to BARC 3b and lower than BARC 3c bleeding (RR 0.22; 95% CI 0.13–0.36; *p *< 0.001) [4]. This study underlines the fact that bleeding events may carry the same or even a stronger prognostic impact than hard ischemic events, such as spontaneous MI. The prognostic relevance of bleeding as compared with ischemic events has been also confirmed by a recent meta-analysis including 16 studies involving >100,000 patients [23].

**Fig. 3.**
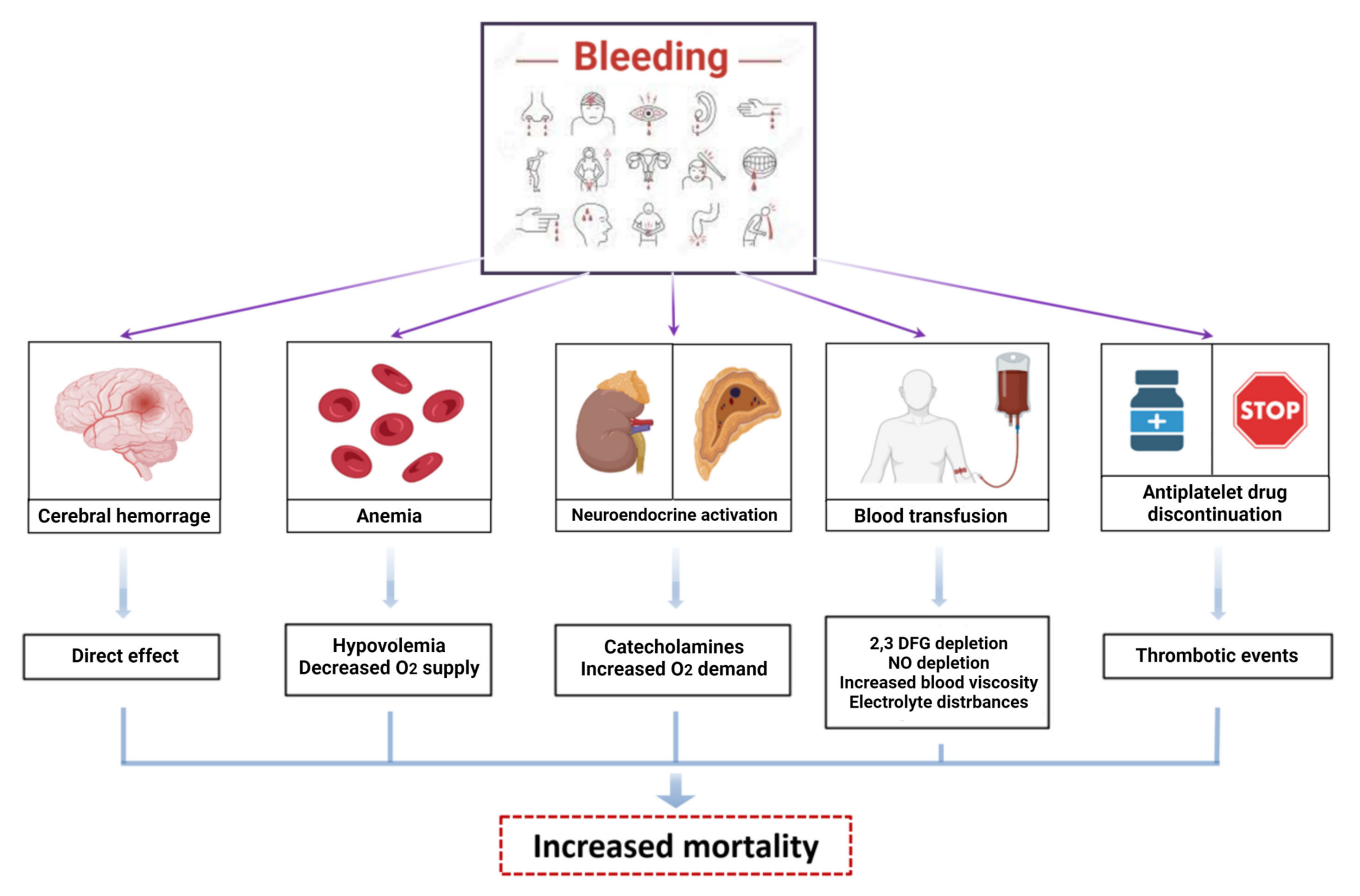
**Mechanisms of damage of bleeding**. Bleeding increases mortality and morbidity in patients treated with antithrombotic therapies after PCI. Multiple mechanisms can explain the adverse outcomes related to bleeding: direct cerebral injury in case of intracranial hemorrhage, anemia and hypovolemia in case of huge losses leading to decreased $O_{2}$ supply to tissues; neuroendocrine activation with release of catecholamines and increased $O_{2}$ demand; depletion of 2,3-diphosphoglyceric acid, nitric oxide and electrolyte disturbances caused by blood transfusions, which reduces $O_{2}$ release at the tissue level and favours vasoconstriction and platelet aggregation; enhanced ischemic risk caused by withdrawal of antithrombotic therapy. Abbreviations. NO, nitric oxide; 2,3 DFG, 2,3-diphosphoglyceric.

Multiple mechanisms may be responsible for the adverse outcomes associated with bleeding, apart from the direct consequences of massive bleeding on hemodynamic status or tissue injury in case of intracranial bleeding (Fig. 3).

Bleeding, including minor, is associated with a higher rate of ischemic events, mainly related to the unplanned interruption of antiplatelet treatment in response to bleeding [24]. Furthermore, activation of coagulation and inflammatory pathways are a physiological counter-response to blood loss, increasing the risk of thrombotic events and atherosclerotic plaque destabilization [25]. Moreover, blood transfusion used to correct severe and acute anemia is associated to a three-fold increase of 30-day mortality, caused by depletion of 2–3 diphosphoglycerate (DPG) and nitric oxide, which reduce tissue oxygen delivery and lead to vascular constriction and platelet aggregation, respectively [26].

In conclusion, bleeding complications after PCI are associated with a rise in the rates of short- and long-term death, non-fatal spontaneous MI, stroke, blood transfusions, longer hospital stay and re-hospitalisation.

## 5. Prevention of Bleeding Complications

### 5.1 Risk Stratification

Identifying clinical and procedural features associated with HBR is essential to define patients with HBR, allowing prompt application of targeted bleeding avoidance strategies and standardized bleeding risk across trials [5]. Several scores have been developed to predict major bleeding. They can be divided according to the setting (in-hospital vs. out-of-hospital), validation cohort, type of events (bleeding events only vs. ischemic and bleeding events) and the approach (semi-quantitative vs. quantitative) (Table 1).

**Table 1.** **Risk stratification scores**.

	PRECISE DAPT score	DAPT score	CRUSADE score	ARC-HBR criteria
Validation	Pooled analysis of 8 randomized trials (n = 14,936)	DAPT randomized trial (n = 11,648)	Registry of high-risk patients with NSTEMI (n = 71,277)	Consensus of experts
				(subsequent validations)
Bleeding outcome	Out-of-hospital bleeding	Major bleeding between 12 and 30 months after PCI	In-hospital major bleeding	Out-of-hospital major bleeding
Bleeding definition used	TIMI major and minor	GUSTO moderate and severe	Crusade major bleeding	BARC major bleeding
Score threshold	Score ≥25	Score –2 to 0	Score ≥50	1 major criterion or 2 minor criteria
Ischemic risk evaluation	No	Yes	No	No
Score range	0 to 100	–2 to 10	0 to 100	Qualitative
Clinical setting	Stable and unstable patients undergoing PCI	Stable and event-free patients 12 months after PCI	NSTEMI	Stable and unstable patients undergoing PCI

Several risk stratification scores have been developed to predict major bleeding events in the past years. Among these, guidelines recognize the PRECISE-DAPT, DAPT and CRUSADE scores and the ACR-HBR criteria. Scores can be divided according to in-hospital vs. out-of-hospital setting, validation cohort, type of predicted events (bleeding only vs. both ischemic and bleeding events), bleeding definition used, approach (semi-quantitative vs. quantitative) and the clinical setting (i.e., CCS and/or ACS). Abbreviations. PCI, percutaneous coronary intervention; DAPT, dual antiplatelet therapy; NSTEMI, non-ST elevation myocardial infarction; CCS, chronic coronary syndrome; ACS, acute coronary syndrome.

For in-hospital bleeding, the “Can Rapid risk stratification of Unstable angina patients Suppress ADverse outcomes with Early implementation of the ACC/American Heart Association (AHA) guidelines” (CRUSADE) score is considered the most discriminatory among NSTEMI patients [27, 28]. It takes into account hemodynamic parameters at presentation (heart rate, systolic blood pressure, heart failure), laboratory findings (hematocrit, creatinine clearance) and clinical features (sex, history of diabetes mellitus or vascular disease). The sum of the weighted integers ranges from 1 to 100 points with a threshold of 50 points, above which the risk of in-hospital major bleeding is considered high [27].

The “PREdicting bleeding Complications In patients undergoing Stent implantation and subsEquent Dual Anti Platelet Therapy” (PRECISE-DAPT) score was introduced to predict the risk of out-of-hospital major bleeding at 1 year [29]. It is applicable at discharge to CCS or ACS treated with PCI and treated with DAPT and no OAC and includes both clinical and laboratory features [30]. HBR patients are defined as having a PRECISE-DAPT score ≥25. In these patients, prolonged DAPT (12–24 months) was associated with no ischemic benefit but rather with a consistent bleeding risk [30].

In 2019, The Academic Research Consortium for High Bleeding Risk (ARC-HBR) proposed a definition encompassing patients having a BARC type 3 or 5 bleeding risk >4 % or an intracranial hemorrhage risk >1 % at 1 year. Of 14 major and 6 minor criteria identified, the presence of at least 1 major or 2 minor criteria enables the definition of HBR. Several studies validated the ARC-HBR definition, suggesting that it performs better than other contemporary bleeding risk scores (Table 2) [31]. More recently, the ARC developed a trade-off model aiming to predict the absolute and relative risks of bleeding and MI and/or ST at the time of PCI, contributing to clinical decision-making for individual patients at HBR.

**Table 2.** **Validation of the ARC-HBR criteria**.

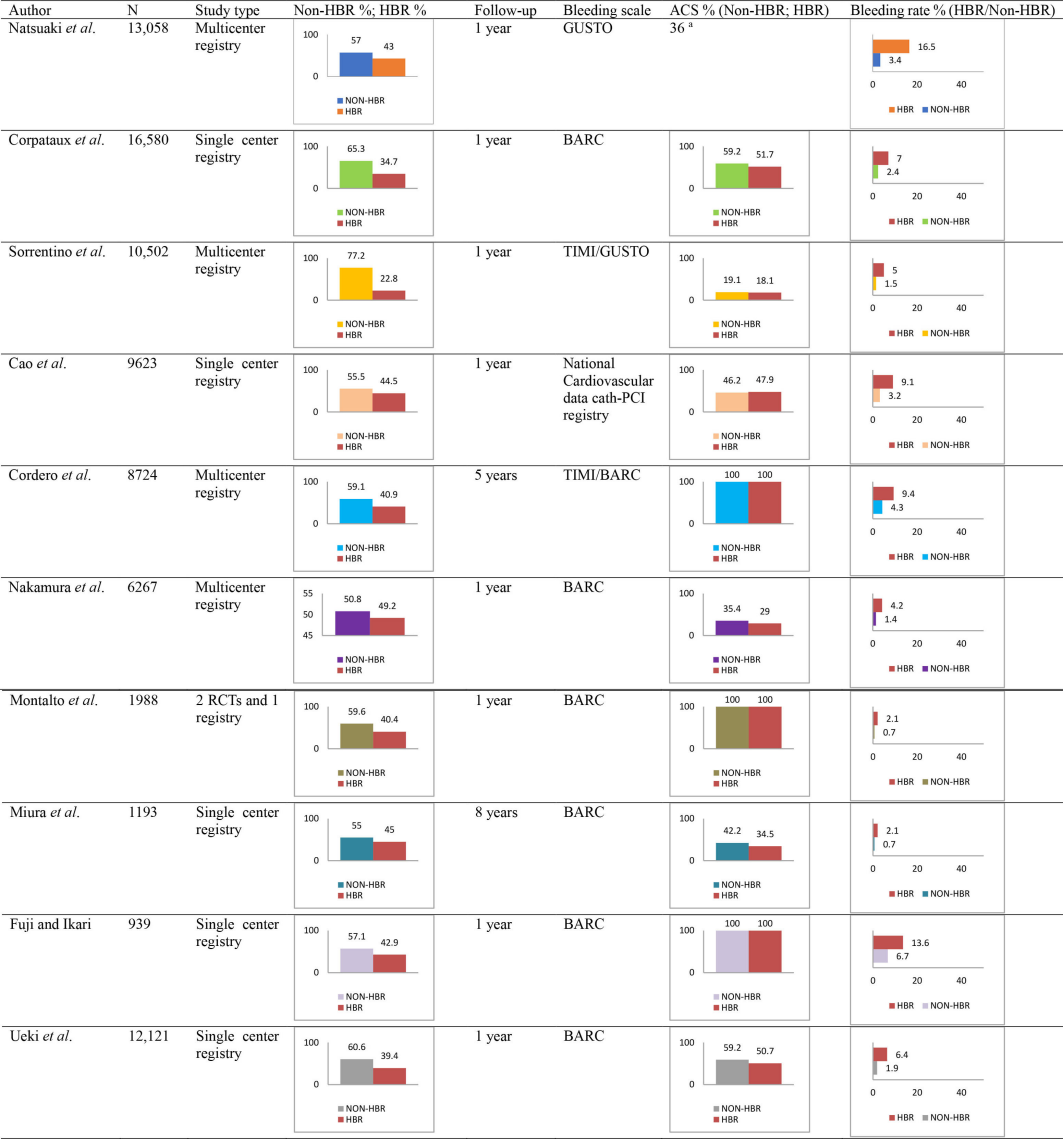

List of all the studies reporting the occurrence of major bleeding according to ARC-HBR status in patients undergoing PCI. Abbreviations: ACS, Acute coronary syndrome; BARC, Bleeding Academic Research Consortium; GUSTO, Global Use of Strategies to Open Occluded Arteries; ARC, Academic Research Consortium; HBR, High Bleeding Risk; RCT, Randomized Clinical Trial; TIMI, Thrombolysis in Myocardial Infarction. ^a^ Stratified data according to ARC-HBR status are not available.

Among risk scores predicting out-of-hospital thrombotic and bleeding events, the DAPT score aims at identifying patients that would benefit from a DAPT prolongation beyond 1 year after PCI [32]. A high-risk score (>2) identifies patients who may benefit from reduced cerebrovascular events with prolonged DAPT, with only a modest increase in bleeding risk, whereas a risk score <2 identifies patients who would be exposed to a significant increase of bleeding without any benefit from DAPT prolongation in terms of reduction of ischemic events. Importantly, bleeding and ischemic risk scores require additional resources, are time-consuming and the majority of them present C-statistics which are sub-optimal (<0.70) and typically lower than those observed in the development cohort (i.e., >0.70), questioning their role in clinical decision making [33, 34, 35, 36].

Besides scores, a number of demographic (i.e., Asian ethnicity or elderly), clinical (i.e., cardiogenic shock or cardiac arrest, previous bleeding, anemia, reduced platelet count, prior stroke, malignancy, severe liver disease, fragility) and procedural (i.e., non-radial access, periprocedural antithrombotic therapy, use of mechanical support) features are associated with increased bleeding risk and should be considered for a correct stratification of individual patient’ bleeding risk (Graphical abstract) [37, 38, 39].

### 5.2 Bleeding Avoidance Strategies

#### 5.2.1 In-Hospital

5.2.1.1 Periprocedural Anticoagulant Therapy

Periprocedural anticoagulation in patients undergoing PCI includes low molecular weight heparin (LMWH), fondaparinux, unfractionated heparin (UFH) or bivalirudin. The latter is recommended only for specific clinical scenarios because of high costs and higher rates of stent thrombosis compared with heparin [27, 32, 33, 40]. With regards to intraprocedural anticoagulation, UFH represents the standard of care regardless of the clinical setting, despite no trial has ever shown its efficacy versus placebo. The only exception to this general rule is the use of bivalirudin in patients with heparin-induced thrombocytopenia [33]. Anticoagulation before and after PCI is typical of the ACS setting and the regimens vary between STEMI and NSTEMI. Before PCI, the anticoagulant associated with the best performance in terms of safety and efficacy is fondaparinux in NSTEMI, while in STEMI it is debated whether LMWH or UFH performs better [40]. Given that UFH is the anticoagulant of choice during PCI and that crossovers between heparins is discouraged, the anticoagulant should be selected also according to practical considerations related with the timing of PCI. In fact, there is no reason for administering an anticoagulant different from UFH in a patient scheduled to undergo PCI in few hours, regardless of clinical presentation [40]. The benefit of UFH pre-treatment in the STEMI setting has been shown in a sub-analysis of the TASTE trial including 7144 patients [41]. In this study, patients pre-treated with UFH less often presented with intracoronary thrombus (61.3% vs. 66.0%, *p *< 0.001) and total vessel occlusion (62.9% vs. 71.6%, *p *< 0.001), compared with those not pre-treated [41]. Moreover, the effect of UFH, but not of LMWH or fondaparinux, can be easily monitored with the activated clotting time (ACT) and reverted by protamine sulfate, making its use safer and more user friendly. Notably, fondaparinux showed different performances in NSTEMI compared to STEMI patients [33]. The OASIS-5 trial has shown a 48% reduction of major bleeding at 9 days among 20,078 NSTEMI patients undergoing invasive treatment in average 2.5 days after hospital admission, compared with LMWH [42]. Conversely, OASIS-6 showed that fondaparinux compared with UFH or placebo was associated with increased rates of guiding catheter thrombosis and more coronary complications, such as abrupt coronary artery closure, new angiographic thrombus, catheter thrombus, no reflow, dissection, or perforation device related thrombosis in STEMI patients, requiring full dose intraprocedural anticoagulation with UFH at the moment of PCI and leading to its contraindication in this setting [33, 43].

With respect to the post-PCI setting, anticoagulation is generally not recommended after PCI, with the exception of ACS patients at high thrombotic burden or those not eligible for coronary revascularization, in whom anticoagulation in-hospital may be considered up to 7 days [44, 45]. In this scenario, the use of fondaparinux may represent the safest option, also in light of the fact that patients are already treated with concomitant DAPT [44].

5.2.1.2 Periprocedural Antiplatelet Therapy

Aspirin is considered the backbone of antiplatelet therapy in patients undergoing PCI, regardless of clinical setting (acute or chronic), in association with a ${\mathrm{P2Y}}_{12}$ receptor inhibitor (clopidogrel, prasugrel or ticagrelor). In ACS, potent ${\mathrm{P2Y}}_{12}$ inhibitors (prasugrel and ticagrelor) are recommended over clopidogrel in patients without contraindications to these medications or HBR, while clopidogrel represents the standard of care for stable patients undergoing PCI [29, 32, 33]. Nevertheless, the most appropriate timing for the administration of oral ${\mathrm{P2Y}}_{12}$ receptor inhibition has represented a topic of great debate over the years. The rationale for the so called “pre-treatment”, consisting in administrating oral ${\mathrm{P2Y}}_{12}$ receptor inhibitor before PCI, is to account for the delay from oral administration to onset of action (ranging from 2 to 6 hours), allowing adequate platelet inhibition at the time of PCI [46]. This strategy may theoretically be associated with reduced risk of early ischemic events such as periprocedural MI, distal coronary embolization and acute ST [47, 48]. However, this theoretical advantage comes at the expenses of a certain increase of bleeding [44, 49]. Routine ${\mathrm{P2Y}}_{12}$ inhibitor pre-treatment is currently recommended by guidelines in STEMI but not in stable patients and NSTEMI, with the indications in the latter setting having undergone drastic changes in the past years [27, 32, 33].

In STEMI, the recommendation towards ${\mathrm{P2Y}}_{12}$ inhibitor pre-treatment stems from the fact these patients present a high thrombotic burden, the surgical option for the acute treatment of MI in these patients has been almost completely abandoned and the onset of action of oral ${\mathrm{P2Y}}_{12}$ inhibitors is particularly delayed (i.e., up to 8 hours) [50, 51]. The ATLANTIC trial showed that a pre-treatment with ticagrelor on average 30 minutes before primary PCI appeared to be safe, with no difference in the rate of major bleeding, but it did not improve pre-PCI coronary reperfusion, compared to ticagrelor, at the moment of PCI. There was a reduction of acute and sub-acute ST in the pre-treatment group, despite the incidence of such events was overall very low (0% vs. 0.8% in the first 24 hours; 0.2% vs. 1.2% at 30 days) [52]. It may be argued that the typically short time from diagnosis to coronary angiography (CA) in STEMI patients, the fact that patients with suspected STEMI may not present a type I MI but other conditions in which potent platelet inhibition is not beneficial (i.e., Takotsubo syndrome, pericarditis/myocarditis, aortic or coronary dissection, epicardial artery or microvascular spasm) and the increasing availability of intravenous antiplatelet agents such as cangrelor or glycoprotein IIb/IIIa inhibitors (GPIs) allowing for a bridging of platelet inhibition once coronary anatomy is known, filling the gap between oral ${\mathrm{P2Y}}_{12}$ inhibitor administration and onset of action, makes the use routine use of ${\mathrm{P2Y}}_{12}$ inhibitor pre-treatment more controversial [53, 54, 55]. Moreover, intravenous antiplatelet agents have the advantage of overcoming the reduced intestinal absorption of oral antiplatelet agents occurring during concomitant opioid administration [56]. Nevertheless, the use of GPIs on top of DAPT—the so called adjunctive antiplatelet strategy—is only recommended for bailout situations such as high thrombotic burden or slow coronary flow, being associated with increased bleeding risk [33]. Further trials are needed to shed light on the safety and efficacy of a routine ${\mathrm{P2Y}}_{12}$ inhibitor pre-treatment in the contemporary STEMI management.

With regards to NSTEMI, a substantial proportion of these patients (up to 30%) do not undergo revascularization or undergo CABG after CA, making routine ${\mathrm{P2Y}}_{12}$ inhibitor pre-treatment potentially harmful and increasing hospital stay and costs [57]. Furthermore, NSTEMI patients have less thrombotic burden, are on average older and with more comorbidities, and therefore at higher bleeding risk, compared to STEMI patients [57]. Moreover, in NSTEMI patients, randomized controlled trials (RCTs) did not show encouraging results on ${\mathrm{P2Y}}_{12}$ inhibitor pre-treatment. ACCOAST found pre-treatment with prasugrel (on average 4 hours before PCI) to increase bleeding without any significant benefit in ischemic events compared with prasugrel at the moment of CA [58]. ISAR-REACT 5 found a strategy with ticagrelor in average 1 hour before PCI was associated with increased ischemic events compared with a strategy of prasugrel at the time of PCI and DUBIUS, despite being largely underpowered, did not find any differences between pre-treatment with ticagrelor (in average 23 hours before PCI) versus ticagrelor or prasugrel at the moment of PCI [59, 60]. Therefore, avoiding routine ${\mathrm{P2Y}}_{12}$ inhibitor pre-treatment in NSTEMI represents a valuable and safe bleeding reduction strategy.

Finally, in stable patients in whom the risk of thrombotic events is lower compared to ACS, the rationale as well as the evidence in support of ${\mathrm{P2Y}}_{12}$ inhibitor pre-treatment is limited [32]. Cangrelor may represent a safe and effective option for stable patients undergoing complex PCI [53]. Moreover, the use of intravenous aspirin in patients chronically treated with clopidogrel has been proposed as a simple but reasonable option to provide rapid and safe platelet inhibition by DAPT without increasing bleeding events [61].

5.2.1.3 Vascular Access Site

Among in-hospital bleeding avoidance strategies, the optimal choice of vascular access site is well known to play a key role. Three large RCTs, have strongly demonstrated a reduction of access site-related bleeding, access site-vascular complications and cardiac mortality with radial compared to femoral access site [62, 63, 64]. Therefore, radial access is now considered the standard of care [1, 27, 32, 33]. Nevertheless, in those situations in which femoral artery may be the only available access, a variety of methods have been developed to reduce access site-vascular complications. These include the optimization of femoral artery puncture by using fluoroscopy and ultrasounds or the micro-puncture technique [65]. The optimization of haemostatic process is also fundamental for reducing bleeding risk and vascular complications at puncture site. Femoral artery haemostasis can be obtained with either manual compression or vascular closure devices, with recent studies suggesting improved outcomes with the use of active closure systems [66]. Therefore, the use of vascular closure devices is highly recommended to reduce bleeding, especially for large bore access.

5.2.1.4 Stent Choice and Optimization

After stent implantation, DAPT is required to avoid local ischemic events such as ST until endothelial coverage occurs [2]. Because different rates of ST have been associated with different stent platforms, the stent choice may impact bleeding risk and DAPT durations after implantation [67, 68]. An emblematic example is the recommended DAPT duration after bare metal stent (BMS) implantation in stable patients is one month' while it was 12 months after first-generation drug eluting stent (DES) implantation given the increased risk of ST with these earlier DES [29].

The limitation of first-generation DES has led to the development of second-generation DES characterized by smaller strut thickness and reduced thrombogenicity [68]. RCTs and single-group studies using historical cohorts as controls have compared the performance of different stent platforms in the setting of short DAPT durations (1 to 6 months versus 12 months) [69, 70, 71, 72, 73, 74]. RCTs represent the highest level of evidence while the latter should be interpreted in light of their methodological limitations (non-randomized design). Among the RCTs, LEADERS FREE compared a drug-coated-stent (BioFreedom, Biolimus A9) versus BMS among 2466 patients at high risk of bleeding (57% CCS, 43% ACS) undergoing 1 month of DAPT and found the former to reduce the primary composite endpoint of cardiac death, MI, or ST (HR 0.71; 95% CI 0.56–0.91; *p* = 0.005) [69]. Similarly, SENIOR showed a 29% reduction of the primary composite endpoint of all-cause mortality, MI, stroke, or ischaemia-driven target lesion revascularization with an everolimus-eluting, biodegradable polymer stent (Synergy, Boston Scientific) compared with BMS in 1200 older patients (55% CCS, 45% ACS) receiving a short duration of DAPT (1 month for CCS and 6 months for ACS) [70]. Finally, Onyx ONE found that the current-generation zotarolimus-eluting, durable polymer stent (Resolute Onyx, Medtronic) was non-inferior to BioFreedom DES in 1996 patients (48% CCS, 51% ACS) at high risk of bleeding treated with 1 month-DAPT-regimen, with regards to the primary composite endpoint including death from cardiac causes, MI, or definite or probable ST at 1 year [71].

Furthermore, non-randomized studies using historical cohorts as controls of patients treated with DES have suggested the safety of the following stent platforms in the setting of short DAPT (1–3 months): Ultimaster (Terumo), Xience (Abbott Vascular) and Synergy (Boston Scientific) [72, 73, 74]. Based on the available data, regulatory agencies have approved the use of Resolute Onyx, Synergy, Xience in the United States and of these platforms plus BioFreedom and Ultimaster in Europe, for patients at high risk of bleeding requiring short DAPT duration.

Regardless of stent platform, there are technical aspects in the setting of PCI that can deeply influence not only stent-related adverse events, including ST and restenosis, but also bleeding. Indeed, the use of double stents for the treatment of bifurcation lesions, the occurrence of edge dissections after stent implantation and stent malapposition and underexpansion, are associated with increased risk of ischemic events, that can be at least partially outweighed by a more intense antiplatelet therapy, which, in turn, increases the risk of bleeding [75, 76]. To this extent, intravascular imaging (intravascular ultrasound, IVUS, and optical coherence tomography, OCT) to guide PCI can reduce the risk of cardiovascular death and major adverse events (MACE) compared with angiography guided PCI [77, 78, 79]. A pioneering study by Colombo *et al*. [80], published in 1995, showed that among patients in which IVUS-guided stent optimization after PCI was performed, the rate of ischemic events was very low despite the implementation of low-intensity antithrombotic regimens (ticlopidine plus aspirin for 5 days or aspirin alone, both without periprocedural anticoagulation). Therefore, refraining from PCI in the absence of prognostic or symptomatic benefits, using last generation stent platforms with best stent optimization, reduced number of implanted stents and reduced technical complexity when possible (i.e., use of provisional rather than double stenting for bifurcation lesions) may reduce the risk of thrombotic events and allow for less intense and shorter antithrombotic regimens, reducing bleeding (Graphical abstract).

5.2.1.5 Special Clinical Settings

5.2.1.5.1 Mechanical Circulatory Support

Mechanical circulatory supports, such as intra-aortic balloon pump, IMPELLA®, and venoarterial extracorporeal membrane oxygenation are increasingly adopted for PCI patients requiring urgent hemodynamic support during ACS or for elective patients undergoing high-risk PCI [81].

Despite providing important hemodynamic benefits (i.e., left ventricular unloading, increased cardiac output, reduced afterload, and increased blood pressure), there is growing evidence supporting the risk of associated complications, particularly systemic and access-related bleeding [82]. Therefore, an appropriate patient selection is needed to reduce the risk of adverse events related to the use of these devices [83].

5.2.1.5.2 PCI after Fibrinolysis

Despite primary PCI is considered the standard of care for STEMI patients, fibrinolysis is recommended when PCI is not feasible within 120 minutes from diagnosis [33]. PCI may be performed after fibrinolysis in three different scenarios: (i) rescue-PCI (r-PCI), that is performed immediately after unsuccesful fibrinolysis; (ii) facilitated PCI (f-PCI), that is performed immediately after successful fibrinolysis (a strategy not recommended by guidelines); and (iii) early (<24 hours) after successful fibrinolysis [33].

Among patients undergoing fibrinolysis, the recommended antithrombotic therapy is represented by aspirin, clopidogrel and parenteral anticoagulation, given until revascularization if performed, or for the duration of hospital stay, up to 8 days [33]. The anticoagulant of choice is represented by enoxaparin, followed by UFH [31]. Fondaparinux may be considered only in STEMI patients treated with streptokinase [33]. The administration of a GPI is not recommended in this setting because it may increase bleeding without improving clinical outcomes. Ticagrelor, prasugrel and bivalirudin have not been extensively studied in STEMI patients treated with fibrinolysis, therefore they do not represent a safe option [33]. The addition of oral antiplatelet and parenteral anticoagulant drugs on top of fibrinolysis may come at the expenses of increased bleeding, which may be exponentially increased in patients undergoing PCI soon after fibrinolysis, because of the need for intraprocedural heparin on top of the antithrombotic cocktail already administered [84].

The REACT trial randomized 427 STEMI patients undergoing thrombolysis to either repeated thrombolysis, conservative management or r-PCI, the latter was associated with reduced MACE (death, recurrent MI, or severe heart failure) and cerebrovascular events, but at the cost of increased minor bleeding (28% versus 3% in the repeated thrombolysis and 0%, in the conservative therapy arm), without, however, any significant difference among groups in major bleeding events [85].

Notably, studies testing f-PCI found that PCI early after fibrinolysis is associated with higher rates of major bleeding, including hemorrhagic stroke, and higher rates of death, indicating that PCI early after fibrinolysis may significantly increase bleeding [86, 87, 88].

In conclusion, PCI early (<6 hours) after fibrinolysis should be considered only in case of unsuccessful fibrinolysis and intra-procedural antithrombotic therapy should be used wisely, taking into account the antithrombotic cocktails administered in the previous hours. Among patients with successful fibrinolysis, PCI should be performed within 24 hours as per guideline recommendations; 12 to 24 hours after fibrinolysis may be preferable to balance efficacy against bleeding risk [33].

5.2.1.5.3 Concomitant Anticoagulation

Between 10% and 15% of patients undergoing PCI is on treatment with oral anticoagulation, raising concerns over the optimal timing of interruption of this therapy or additional intraprocedural anticoagulation [89]. This dilemma is particularly true among patients requiring an urgent invasive strategy [27, 32]. Indeed, in case of elective PCI, the procedure can be delayed until the effects of OAC have waned off, but such delay is not possible for ACS patients undergoing urgent or emergent CA. For elective PCI, the recommendation is to wait for an INR ≤2 for radial access and ≤1.5 for femoral access among patients treated with VKA and from 24 to 96 hours for patients treated with NOAC, depending on the NOAC used, renal function and bleeding risk of the procedure [90]. For urgent or emergent PCI, the consensus is to administer a regular dose of intraprocedural UFH regardless of last OAC intake, prefer primary PCI over fibrinolysis regardless of the anticipated time to PCI-mediated reperfusion, avoid the use of GPI and use clopidogrel as the ${\mathrm{P2Y}}_{12}$ inhibitor of choice [90].

#### 5.2.2 Out-of-Hospital 

**Fig. 4.**
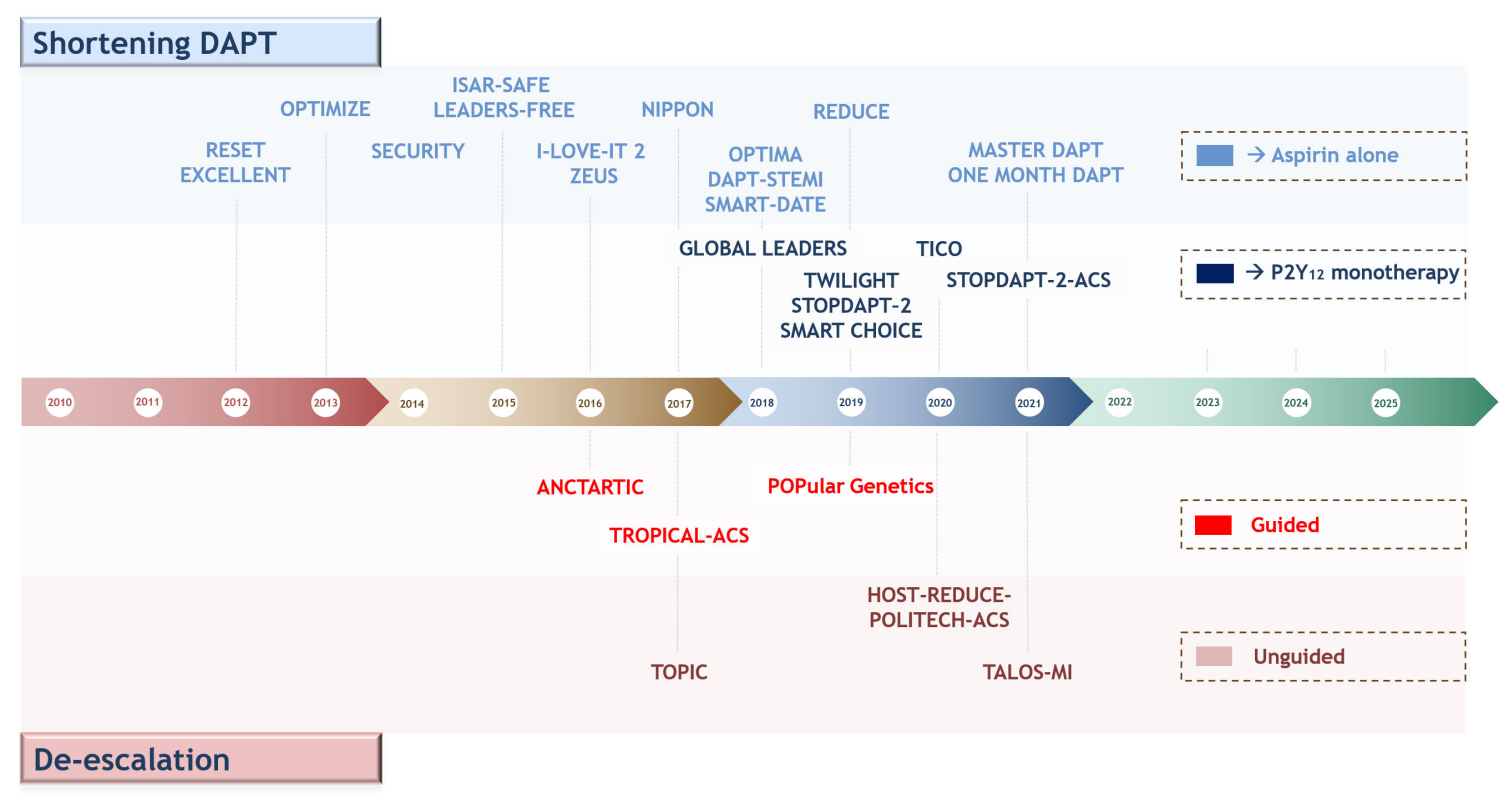
**Timeline of randomized controlled trials testing antiplatelet regimens aiming at reducing bleeding events after percutaneous coronary interventions or acute coronary syndrome**. Upper: shortening DAPT and aspirin-free strategies; lower: guided and un-guided DAPT de-escalation. Abbreviations. DAPT, dual antiplatelet therapy.

The availability of stent platforms with less thrombogenic profiles, together with the increasing understanding of the prognostic relevance of bleeding events and the fact that ischemic and bleeding risks may vary over time, with the former being highest during the first months after PCI and the latter remaining relatively stable over time, has fuelled the interest towards antithrombotic strategies aiming at reducing the incidence of bleeding without any trade-off in ischemic events among patients who have undergone PCI [2] (Fig. 4). Furthermore, there is increasing evidence supporting differences in individual responsiveness to ${\mathrm{P2Y}}_{12}$ inhibitors may carry important clinical implications and should be considered when tailoring antiplatelet therapy in patients with ACS and/or undergoing PCI, renewing the interest towards the use of tailored antiplatelet therapy [91, 92].

5.2.2.1 Shortening DAPT Duration

The first and most largely adopted strategy in this setting consists in shortening DAPT duration (1 to 6 months), followed by the use of aspirin alone. This strategy has been evaluated in thirteen RCTs, of which seven compared 6-month versus 12-month DAPT, four trials included 3-month versus 12-month DAPT and two a one-month versus 6- and 12-months DAPT [2]. Overall, individual RCTs and pooled analyses have shown shortening DAPT duration may reduce bleeding without any trade-off in ischemic events in patients with CCS, while the abrupt shortening of DAPT duration in ACS patients may be associated with an increase in ischemic events. The, SMART-DATE trial, randomized 2717 ACS patients (38% STEMI, 31% NSTEMI and 31% unstable angina) to either 6-month or 12-month DAPT (mainly using clopidogrel as ${\mathrm{P2Y}}_{12}$ inhibitor), despite meeting the primary endpoint for non-inferiority for all-cause death, MI, or stroke at 18 months, there was an higher rate of MI (HR 2.41; 95% CI 1.15–5.05; *p* = 0.02) within the 6-month DAPT group [93]. On this background, current international guidelines recommend a standard DAPT duration of 6 months for CCS and 12 months for ACS, but DAPT may be shortened up to 1 or 3 months in CCS or NSTEMI and up to 6 months in STEMI among patients at high bleeding risk [1, 27, 33]. Finally, the recent MASTER DAPT trial, showed that 1-month DAPT resulted in a lower incidence of major or clinically relevant non-major bleeding without any difference in NACE or MACE compared with the continuation of therapy for at least 2 additional months [94].

5.2.2.2 ${\mathrm{P2Y}}_{12}$ Monotherapy after a Short Course of DAPT

Pharmacodynamic (PD) investigations showing that aspirin provides limited antithrombotic effects in addition to potent ${\mathrm{P2Y}}_{12}$ blockade have supported the use of ${\mathrm{P2Y}}_{12}$ inhibitor monotherapy approaches [95, 96]. Therefore, in the attempt to provide a better balance between ischemic and bleeding risks compared to a strategy of short DAPT, ${\mathrm{P2Y}}_{12}$ inhibitor monotherapy after a short course of DAPT in patients undergoing PCI has been tested in a number of RCTs [97]. These trials may be distinguished according to the ${\mathrm{P2Y}}_{12}$ inhibitor used as monotherapy. STOPDAPT-2 and SMART-CHOICE tested a strategy of clopidogrel monotherapy after 1 month and 3 month DAPT, respectively, and found this strategy to reduce bleeding and non-inferior to standard 12 months DAPT in terms of composite ischemic events [98, 99]. Nevertheless, these trials enrolled both ACS and CCS patients and had non-inferiority designs, failing to reassure on the safety of such strategy among ACS patients with respect to hard individual endpoints [98, 99]. Indeed, the subsequent STOPDAPT-2 ACS trial, which compared a 1 to 2 month DAPT followed by clopidogrel monotherapy versus standard 12 month DAPT among ACS patients, failed to meet the non-inferiority for the primary composite endpoint of net adverse clinical events (NACE), including cardiovascular death, MI, ST, stroke and TIMI major/minor bleeding [100]. Therefore, despite the use of clopidogrel monotherapy reduces bleeding compared to a standard DAPT, this strategy should be used with caution in patients with ACS or in those at high ischemic risk. With respect to trials using ticagrelor monotherapy early (1–3 months) after a short course of DAPT, these found an overall good performance of this strategy compared with standard 12 month DAPT [101]. In fact, ticagrelor monotherapy 1–3 months after PCI reduced the risk of bleeding without any trade-off in MACE including in patients with ACS [101, 102]. The use of prasugrel in the setting of free-aspirin strategies is limited, being investigated so far only in a pilot single-arm study [103].

The fact clopidogrel but not ticagrelor monotherapy after a short course of DAPT has been associated with increased ischemic events in ACS is consistent with the well-known higher risk of ischemic events of ACS patients compared to CCS patients and by the fact that about 30% of patients treated with clopidogrel, but less than 5% of those treated with ticagrelor (or prasugrel), result in inadequate platelet inhibition leading to high platelet reactivity (HPR), a modifiable marker of thrombotic risk [91, 92]. This difference in the PD response to clopidogrel is related to the fact that clopidogrel is a pro-drug that requires a 2-step biotransformation oxidative process by the hepatic cytochrome (CYP) P450 system to be activated. The CYP2C19 enzyme is involved in both metabolic steps of clopidogrel biotransformation and the gene responsible for its transcription is highly polymorphic, with carriers of loss-of-function (LoF) alleles *2 and *3 being associated with reduced generation of clopidogrel’s active metabolite leading to high HPR rates, and increased thrombotic complications [92, 104].

5.2.2.3 De-Escalation of ${\mathrm{P2Y}}_{12}$ Inhibitors

Prasugrel and ticagrelor are characterized by more potent and predictable pharmacodynamic effects compared with clopidogrel, which however lead to an increased risk of bleeding [105, 106]. Switching from a more potent (prasugrel or ticagrelor) to a less potent (clopidogrel) or lower dose ${\mathrm{P2Y}}_{12}$ inhibitor (i.e., prasugrel 5 mg die) is a strategy called “de-escalation” [2]. Since potent ${\mathrm{P2Y}}_{12}$ inhibitors are preferred over clopidogrel in ACS but not in CCS, this strategy mainly applies to ACS patients. De-escalation of ${\mathrm{P2Y}}_{12}$ inhibitors may be either guided or unguided. A guided approach implies the use of platelet function (PFT) or genetic tests that rule out clopidogrel-HPR or the presence of CYP2C19 LoF alleles which are known to be associated with an increased risk of thrombotic complications post-PCI [107, 108]. An unguided approach consists in de-escalation without the aid of platelet function or genetic testing, typically 1–3 months after PCI, which is the period in which the risk of ischemic events is highest.

5.2.2.3.1 Guided De-Escalation

The rationale for the use of a guided de-escalation is to selectively administer a potent ${\mathrm{P2Y}}_{12}$ inhibitor (prasugrel or ticagrelor) to clopidogrel non-responders, reducing the risk of bleeding that would be associated with an unguided use of these more potent antiplatelet agents and, at the same time, overcoming the increased rate of ischemic events associated with clopidogrel non-responsiveness [91, 92]. Therefore, the clinical impact to be expected from a guided de-escalation is a reduction of bleeding without any trade-off in efficacy.

Three RCTs have tested a guided de-escalation strategy, two using PFT and one using genetic test [109, 110, 111]. ANTARCTIC failed to show reduced NACE with PFT-guided de-escalation versus standard therapy in 877 elderly patients with ACS undergoing PCI. Nevertheless, reduced dose (5 mg daily) of prasugrel rather the recommended 10 mg daily was used in this trial, potentially blunting the superior safety of a de-escalation strategy [109]. On the contrary, TROPICAL-ACS met the composite primary endpoint for non-inferiority of NACE in 2610 patients with ACS [110]. Furthermore, POPular Genetics, which randomized to either genotype-guided de-escalation or standard therapy (mainly ticagrelor) 2488 STEMI patients, showed the non-inferiority in the primary endpoint of NACE and a significant 22% reduction in the co-primary endpoint of PLATO major and minor bleeding at 12 months [111]. Nevertheless, the use of a primary endpoint including both ischemic and bleeding outcomes and the non-inferiority design of these two latter trials represent important limitations contributing to the relatively weak recommendations on the use of PFT or genetic guidance in clinical practice (Class IIb, level of evidence A) [27]. Indeed, such trials were not powered for hard, individual, ischemic or hemorrhagic endpoints such as CV death, MI, ST, major bleeding and intracranial hemorrhage. To this extent, meta-analysis are useful to overcome the limited statistical power for rare endpoints. A recent comprehensive meta-analysis overcoming this limitation showed that a guided de-escalation is associated with a 19% reduction of bleeding without any trade-off in ischemic events [112]. Moreover, a network meta-analysis comparing guided de-escalation versus prasugrel or ticagrelor among more than 60,000 ACS patients from 15 RCTs showed guided de-escalation to be associated with the most favorable balance between safety and efficacy [113]. Collectively, PFT or genetic testing represents a promising strategy for reducing bleeding without any trade-off in ischemic events among ACS patients and future guidelines are likely to provide stronger recommendations on the use of a guided selection of ${\mathrm{P2Y}}_{12}$ inhibiting therapy, based on recent evidence.

5.2.2.3.2 Unguided De-Escalation

The rationale for the use of an unguided de-escalation strategy stems from the fact that while ischemic risk decreases after 1 to 3 months post-PCI, bleeding risk, although being higher in the periprocedural phase, tends to be stable over time [2]. Therefore, potent ${\mathrm{P2Y}}_{12}$ inhibitors (i.e., prasugrel and ticagrelor) would play a key role in reducing the high incidence of ischemic events in the early phase, while a less aggressive antiplatelet regimen would provide a reduction of bleeding without a significant increase of ischemic events, 1 to 3 month after PCI [2].

Three RCTs, for a total of 5681 patients have tested an unguided de-escalation 1 month after ACS versus standard 12-month DAPT. In two of them, de-escalation consisted in switching from a potent ${\mathrm{P2Y}}_{12}$ inhibitor (ticagrelor or prasugrel) to clopidogrel and in one de-escalation consisted in a reduction of prasugrel dosage from 10 mg daily to 5 mg daily. Moreover, two out of three of these trials were conducted in East Asian patients. These trials and subsequent pooled analysis found that unguided de-escalation is effective in reducing bleeding without any trade-off in ischemic events [114, 115, 116].

Limitations of the unguided de-escalation of antiplatelet therapy are the following: (1) 5035 of the 5681 patients in which this strategy was tested were East Asian patients, a population in which bleeding events are higher and ischemic events are lower compared to other populations, therefore, further studies are needed before generalization of their results to different populations; (2) further evidence are needed to show how a de-escalation consisting in a dose reduction of potent ${\mathrm{P2Y}}_{12}$ inhibitor compares with a de-escalation to clopidogrel; (3) because the unguided use of clopidogrel 1 month after ACS may be associated with increased ischemic events in patients non-responder to clopidogrel, further studies are needed to provide reassurance of such an unguided use of clopidogrel in high ischemic risk patients undergoing PCI.

In summary, unguided de-escalation is a very effective and promising strategy in reducing bleeding among ACS patients undergoing PCI, but whether this strategy may be broadly adopted regardless of individual response to clopidogrel requires further investigation.

5.2.2.3.3 Special Clinical Scenarios

Patients Requiring Long Term Anticoagulation

Up to 15% of patients undergoing PCI are affected by a concomitant medical condition requiring OAC, among which AF is the most frequent [117]. Because the addition of DAPT to OAC (the so called triple antithrombotic therapy, TAT) increases the risk of bleeding two- to three-fold compared to OAC alone, strategies to reduce bleeding are particularly important in this clinical setting [14]. To this extent, recent guidelines propose the use of NOAC over VKA and the shortening of TAT to one week followed by clopidogrel plus OAC for the majority of patients [118, 119, 120]. These recommendations are based on the evidence of 4 RCTs comparing each of the 4 available NOACs plus a ${\mathrm{P2Y}}_{12}$ inhibitor (mainly clopidogrel) and aspirin for 1–6 days (average of 4 days) followed by NOAC plus clopidogrel alone versus a TAT lasting in average 4.7 months using aspirin, clopidogrel and a VKA [121, 122, 123, 124]. Collectively, these studies found a 36% of major bleeding and a 49% reduction of intracranial hemorrhage with short versus long TAT [125, 126]. However, none of these RCTs were powered to assess individual ischemic outcomes such as MI or ST [127]. In the attempt of overcoming this limitation, several meta-analysis were performed and showed a potential increase in thrombotic complications with a short TAT (i.e., clopidogrel plus NOAC), especially in patients presenting with ACS [125, 127].

Moreover, it may be argued that these trials present important limitations, such as: (1) none of them focused on ACS patients; (2) procedural complexity was not reported; (3) low ischemic patients were included; and (4) the strategy tested does not reflect the clinical question of whether TAT with NOAC lasting one month would be beneficial compared to TAT lasting 7 days [128]. Furthermore, in light of the fact that approximately 30% of patients treated with clopidogrel are non-responders, such an early drop of aspirin could be particularly detrimental. In summary, these data suggest that shortening TAT duration to 7 days may be a very effective strategy in reducing bleeding but caution should be paid with patients at high ischemic risk or those with ACS, in which prolonging TAT with a NOAC for 1 month may represent a more balanced strategy [128].

#### 5.2.3 Additional Strategies

Gastrointestinal (GI) bleeding is the most frequent source of out-of-hospital bleeding after PCI [129]. Several trials have shown that PPIs (proton pump inhibitors) and histamine H2-receptor antagonist reduce the rate of recurrent gastrointestinal bleeding in patients receiving aspirin at high-risk of GI bleeding [130, 131]. On the basis of these results, international guidelines recommend the routine use of PPI in combination with DAPT, regardless of GI bleeding risk [29]. Among PPIs, pantoprazole or rabeprazole should be preferred over others due to their potential interaction with the CYP2C19 which is also implied in clopidogrel metabolism [132].

Additional strategies to reduce the risk of bleeding in patients treated with anti-thrombotic drugs include optimal control of blood pressure and avoidance of non-steroidal anti-inflammatory drugs [133, 134].

## 6. Acute Management of Bleeding

**Fig. 5.**
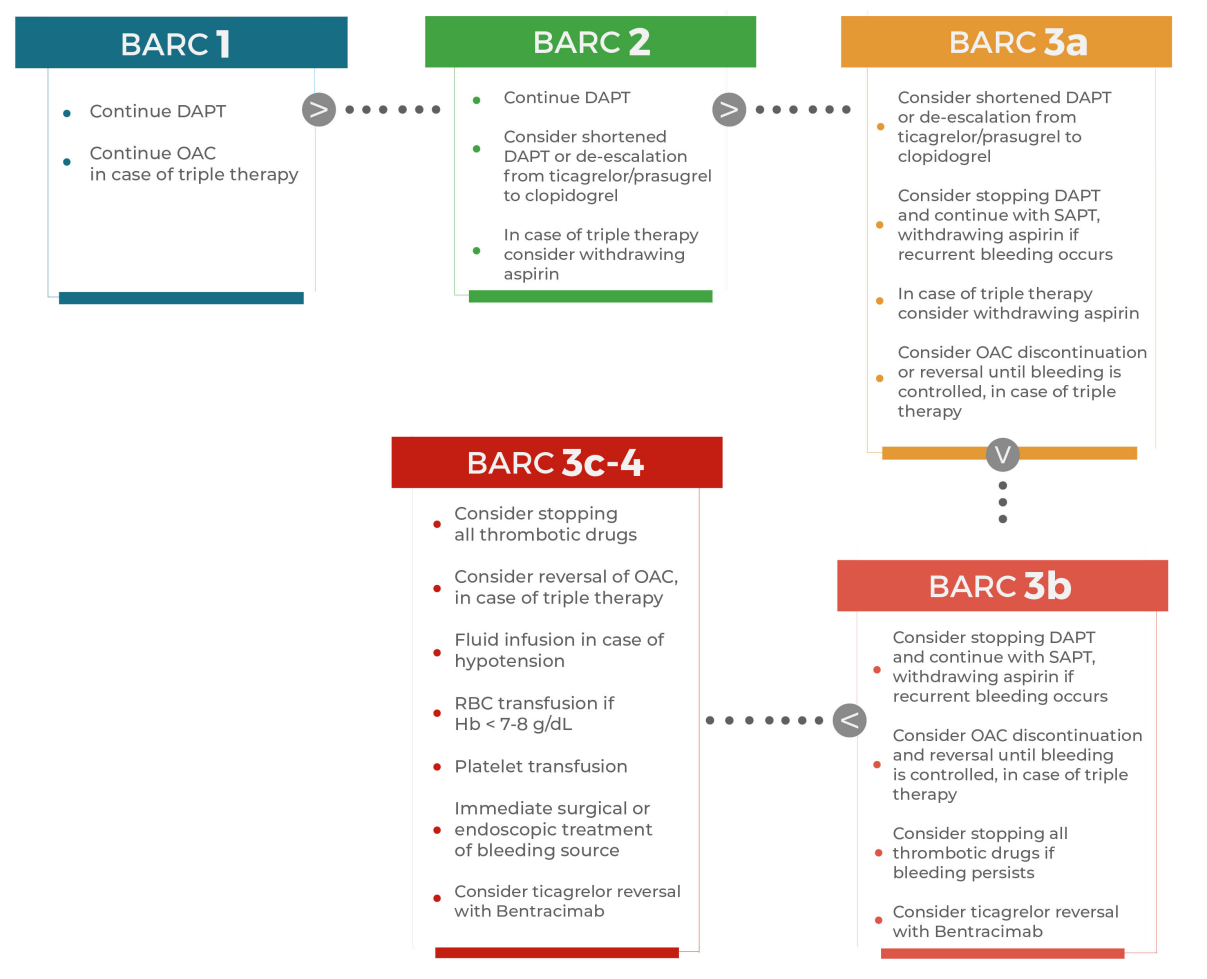
**Practical considerations for the management of bleeding (classified by BARC) in patients treated with dual antiplatelet therapy with or without concomitant oral anticoagulation**. Several strategies can be implemented in case of bleeding, depending on bleeding severity according to BARC classification. BARC 1, BARC 2 and BARC 3a bleeds do not require integrative treatments apart from redefinition of duration and type of antithrombotic treatment (i.e., withdrawal of aspirin or de-escalation of ${\mathrm{P2Y}}_{12}$ inhibitor). On the contrary, BARC 3b, BARC 3c and BARC 4 bleeds require surgical or endoscopic procedures to treat the source of bleeding. In addition, red blood cells transfusion should be considered when hemoglobin levels fall below 7–8 g/dL, although a single cut-off does not exist and every situation must be considered in the light of patient comorbidities and hemodynamic status. In case of major or life-threating bleeding in patients treated with NOACs or ticagrelor, a specific reversal agent should be considered (i.e., idarucizumab for dabigatran, andexanet for edoxaban, apixaban and rivaroxaban, bentracimab for ticagrelor). Abbreviations. DAPT, dual antiplatelet therapy; OAC, oral anticoagulation; SAPT, single antiplatelet therapy; RBC, red blood cells; BARC, Bleeding Academic Research Consortium.

Acute management of bleeding complications in patients treated with antithrombotic therapy is very challenging and scarce evidence is available from RCTs. Therefore, recommendations on acute bleeding management are based on expert opinion or observational studies [90, 135]. Fig. 5 provides a flow chart for the management of bleeding in patients treated with OAC +/ DAPT or SAPT (single antiplatelet therapy) [29].

With respect to non-specific reversal agents for antiplatelet therapies, platelet transfusion or desmopressin have been proposed over years, but none of these is strongly recommended by guidelines [135]. Platelet transfusion has been tested in two RCTs reporting clinical endpoints that have included 970 patients with intracranial hemorrhage taking antiplatelet therapy [136, 137]. These trials showed that platelet transfusion increases the risk of death compared with standard care in patients who do not need a neurosurgical intervention, while it reduces mortality in patients with intracranial hemorrhage undergoing neurosurgery [136, 137]. Desmopressin, has been mainly studied in the setting of elective or emergent cardiac surgery in patients on antiplatelet therapy or affected by platelet dysfunction [138]; it was associated with a 25% reduction in total volume of red blood cells transfused and a 23% reduction in blood loss, as well as with lower risk of re-operation due to bleeding [138]. Nevertheless, there was no decrease in mortality nor increase in thrombotic events with its use [138]. Current guidelines recommend a single dose of desmopressin for intracranial hemorrhage associated with aspirin or ${\mathrm{P2Y}}_{12}$ inhibitors use [135].

Because ticagrelor has reversible binding kinetics and a relatively long half-life (9–12 hours) as opposed to the irreversible binding of aspirin, clopidogrel and prasugrel, platelet transfusion is ineffective in reversing platelet function within 24 hours from ticagrelor withdrawal [139]. To this extent, bentracimab, a recombinant monoclonal antibody fragment that reverses the antiplatelet effects of ticagrelor within 5 minutes has been produced and has been recently tested in the REVERSE-IT trial, among patients undergoing urgent surgery/procedure or with major bleeding. At the interim analysis, bentracimab successfully met the primary reversal endpoint consisting in minimum % inhibition of VerifyNow PRU within 4 hours, with onset of action after 5 minutes of drug initiation [140]. No safety concern emerged from the interim analysis. The trial is still ongoing and completion is expected in 2023.

In the presence of a major or life-threatening bleeding on a VKA, reversal agents are represented by prothrombin complex concentrate, fresh frozen plasma or recombinant activated factor VII [141]. Prothrombin complex is the first choice reversal agent, followed by plasma and factor VII, because it seems to be more effective than plasma in correcting INR, does not require crossmatching, is virally inactivated, does not pose a risk of volume overload and is associated with a lower risk of thrombosis than factor VII, whose use is restricted to cases in which prothrombin complex and plasma are not available [27, 142].

For NOAC-treated patients with intracranial hemorrhage or bleeding involving a critical organ, in case of treatment with dabigatran, first-line treatment is represented by its specific antidote idarucizumab, followed by prothrombin complex concentrates in case of its unavailability [27, 143].

For patients treated with apixaban, edoxaban or rivaroxaban, prothrombin complex concentrate should be first-line treatment [27]. A specific antidote, andexanet, has been developed for factor X inhibitors and evaluated in a single trial, involving 67 patients with acute major bleeding [144]. It reached effective hemostasis in 79% of patients, with no serious side effects [144]. Further studies, with a larger sample size and a control arm, are needed to assess efficacy and safety of this antidote.

## 7. Future Perspectives

A number of strategies may be implemented to reduce bleeding in patients undergoing PCI (Graphical abstract). Among these, a careful selection of patients undergoing PCI, the increasing adoption of more advanced stent platforms and more refined techniques and technologies to optimize stent implantation and the use of more balanced antithrombotic regimens will be key in reducing the risk of bleeding after PCI. It is becoming increasingly clear that a “one-size-fits-all” approach is not successful when selecting antithrombotic therapy in these patients, given to the broad individual response to treatments. Personalization of antithrombotic therapy, taking into account individual ischemic and hemorrhagic risks but also individual responses to antiplatelet agents such as clopidogrel represents the most promising strategy for an optimal balance between bleeding and ischemic prevention at the individual patient’s level [145].

Novel antithrombotic regimens as well as their combinations are currently being tested and may play a key role not only in reducing bleeding but also in reducing the still high rate of ischemic recurrences by promoting plaque stabilization reducing systemic inflammation [146]. Indeed, inflammatory and thrombotic pathways have been shown to be strictly connected and play a key role in the pathogenesis of atherosclerotic disease. To this extent, targeting inflammation on top of antithrombotic drugs (i.e., anti-IL-1β and IL-6 monoclonal antibodies or colchicine) can further reduce thrombotic events without increasing bleeding risk. Furthermore, a strategy of dual-pathway inhibition (DPI), consisting in adding a vascular dose of rivaroxaban (i.e., 2.5 mg twice daily) to a single antiplatelet agent (typically aspirin) has shown promising results in terms of clinical outcomes and reduction of thrombin generation—which plays a key role both in thrombotic and inflammatory pathways—in recent studies, suggesting also the possible effectiveness of a DPI using a ${\mathrm{P2Y}}_{12}$ inhibitor in lieu of aspirin [147, 148, 149].

Another promising line of research is represented by FXIa inhibitors. FXIa has been considered to contribute to thrombosis while playing a relatively minor role in haemostasis. Therefore, its inhibition may potentially lead to reduced ischemic events without increased bleeding [150]. Three compounds are in clinical development: (1) asundexian, a small molecule FXI(a) inhibitor; (2) osocimab, anti-FXI(a) antibody, and (3) fesomersen, a FXI-ligand-conjugated antisense oligonucleotide [151, 152]. Asundexian has recently shown to reduce bleeding without any trade-off in efficacy in a phase II trial comparing different doses versus apixaban among AF patients [153]. Other phase II studies, including over 4000 patients, are ongoing in patients with recent ischemic stroke or recent MI [153].

New formulations of aspirin have been proposed in the attempt to make aspirin more tolerable and reduce bleeding in the GI tract, such as designing enteric-coated aspirin with cellulose or silicon which resists disintegration in the stomach, permitting aspirin to dissolve specifically in the duodenum, avoiding topic epithelial injury [154]. A liquid formulation of a novel pharmaceutical lipid–aspirin complex (PL-ASA) was designed to prevent disruption of protective gastric phospholipid barrier, avoiding direct acid injury and has provided promising results in pharmacokinetic and pharmacodynamic studies [155].

The development of new reversal agents is under way and may be of particular interest for the prompt treatment of bleeding complications among patients treated with antithrombotic agents. Among these, ciraparantag is a small molecule that has been reported to bind all NOACs as well as LMWH and UFH and fondaparinux [156]. Therefore, ciraparantag may potentially function as a universal reversal agent for several classes of anticoagulants, enhancing their safety profile. A phase II RCT is ongoing to evaluate the efficacy and safety of ciraparantag for reversal of anticoagulation induced by different anticoagulant drugs (edoxaban, apixaban or rivaroxaban) in generally healthy adults, whose results are expected in December 2022 [157]. Finally, UHRA-7 is a multivalent polymer designed to be a universal heparin reversal agent (both UFH and LMWH) that is currently being studied in preclinical trials [158].

## 8. Conclusions

For many years, the main concern in patients undergoing PCI has been the reduction of ischemic complications. The increasing awareness that bleeding complications are relatively common and carry important prognostic implications has recently shifted the interest towards the implementation of bleeding reduction strategies. To this extent, prevention represents the most effective and cost-effective strategy. Bleeding prevention strategies include patient bleeding risk stratification, careful assessment of the eligibility for invasive and high-risk procedures, personalized antithrombotic therapy and implementation of advanced stent platforms and procedural techniques. Finally, when a bleeding occurs, prompt and effective treatment is essential and may be achieved by new reversal agents and technologies.

## Abbreviations

ACCOAST, Comparison of Prasugrel at the Time of Percutaneous Coronary Intervention or as Pretreatment at the Time of Diagnosis in Patients with Non-ST Elevation Myocardial Infarction; ACS, Acute Coronary Syndrome; ADAPT-DES, Assessment of Dual AntiPlatelet Therapy With Drug Eluting Stents; AF, Atrial Fibrillation; ANTARCTIC, Platelet function monitoring in elderly patients on prasugrel after stenting for an acute coronary syndrome; ARC-HBR, Academic Research Consortium for High Bleeding Risk; ASA, AcetylSalicylic Acid; ATLANTIC, Administration of Ticagrelor in the Cath Lab or in the Ambulance for New ST Elevation Myocardial Infarction to Open the Coronary Artery; AUGUSTUS, An Open-Label, 2×2 Factorial, Randomized Controlled, Clinical Trial to Evaluate the Safety of Apixaban Versus Vitamin K Antagonist and Aspirin Versus Aspirin Placebo in Patients With Atrial Fibrillation and Acute Coronary Syndrome or Percutaneous Coronary Intervention; BARC, Bleeding Academic Research Consortium; BMS, Bare metal stent; CA, Coronary Angiography; CABG, Coronary Artery Bypass Graft; CCS, Chronic Coronary Syndrome; CRUSADE, Can Rapid risk stratification of Unstable angina patients Suppress ADverse outcomes with Early implementation of the ACC/American Heart Association (AHA) guidelines; CYP, Cytochrome; DAPT, Dual Anti-Platelet Therapy; DAT, Double Antithrombotic Therapy; DES, Drug Eluting Stent; DPI, Dual Pathway Inhibition; DPG, Di-Phosphoglycerate; DUBIUS, Downstream Versus Upstream Strategy for the Administration of P2Y12 Receptor Blockers In Non-ST Elevated Acute Coronary Syndromes With Initial Invasive Indication; ENTRUST AF PCI, Edoxaban Treatment vs. Vitamin K Antagonist in Patients With Atrial Fibrillation Undergoing Percutaneous Coronary Intervention; f-PCI, Facilitated-PCI; GI, Gastrointestinal; GPI, Glycoprotein IIb/IIIa Inhibitors; GUSTO, Global Use of Strategies to Open Occluded Arteries; HBR, High-Bleeding Risk; HP, Helicobacter Pylori; HPR, High-Platelet Reactivity; IHD, Ischemic Heart Disease; ILUMIEN III, Multicenter Randomized Trial of OCT Compared to IVUS and Angiography to Guide Coronary Stent Implantation; INR, International Normalized Ratio; ISAR-REACT, Intracoronary Stenting and Antithrombotic Regimen: Rapid Early Action for Coronary Treatment; ISTH, International Society on Thrombosis and Haemostasis; IVUS, Intra-vascular ultrasound; LoF, Loss of Function; MACE, Major Adverse Cardiovascular Events; MI, Myocardial Infarction; NACE, Net Adverse Clinical Events; NOAC, Non-Vitamin K antagonists Oral Anticoagulants; NSTE-ACS, Non-ST Elevation Acute Coronary Syndrome; OAC, Oral Anti-Coagulation; OASIS-5, Organization for the Assessment of Strategies for Ischemic Syndromes; OASIS-6, Effects of fondaparinux on mortality and reinfarction in patients with acute ST-segment elevation myocardial infarction; OCT, Optical Coherence Tomography; ONYX ONE, A Randomized Controlled Trial With Resolute Onyx in One Month Dual Antiplatelet Therapy for High-Bleeding Risk Patients; PIONEER AF, An Open-Label, Randomized, Controlled, Multicenter Study Exploring Two Treatment Strategies of Rivaroxaban and a Dose-Adjusted Oral VKA Treatment Strategy in Subjects With Atrial Fibrillation Who Undergo Percutaneous Coronary Intervention; PLATO, Platelet Inhibition and Patient Outcomes; PCI, Percutaneous Coronary Intervention; PD, Pharmacodynamic; PDB, Post-Discharge Bleeding; PFT, Platelet Function Tests; POPULAR GENETICS, CYP2C19 Genotype-Guided Antiplatelet Therapy in ST-Segment Elevation Myocardial Infarction Patients — Patient Outcome after Primary PCI; PPI, Proton pump inhibitors; PRECISE-DAPT, PREdicting bleeding Complications In patients undergoing Stent implantation and subsEquent Dual Anti Platelet Therapy; r-PCI, Rescue-PCI; RE-DUAL PCI, Randomized Evaluation of Dual Antithrombotic Therapy With Dabigatran vs. Triple Therapy With Warfarin in Patients With Non-valvular Atrial Fibrillation Undergoing Percutaneous Coronary Intervention; REACT, Rescue Angioplasty versus Conservative Treatment or Repeat Thrombolysis; REVERSE-IT, Rapid and Sustained Reversal of Ticagrelor–Intervention Trial; RCT, Randomized Controlled Trial; SAPT, Single AntiPlatelet Therapy; SENIOR, Drug-eluting stents in elderly patients with coronary artery disease; SMART DATE, 6-month versus 12-month or longer dual antiplatelet therapy after percutaneous coronary intervention in patients with acute coronary syndrome; SMART CHOICE, Safety of 3-Month Dual Antiplatelet Therapy After Implantation of Ultrathin Sirolimus-Eluting Stents With Biodegradable Polymer (Orsiro); ST, Stent Thrombosis; STEMI, ST Elevation Myocardial Infarction; STOP-DAPT 2, Short and Optimal Duration of Dual Antiplatelet Therapy-2; STOP-DAPT-2 ACS, Short and Optimal Duration of Dual Antiplatelet Therapy After Everolimus-Eluting Cobalt-Chromium Stent-2 Acute Coronary Syndrome; TAT, Triple Antithrombotic Therapy; TIMI, Thrombolysis in Myocardial Infarction; TRACER, Thrombin Receptor Antagonist for Clinical Event Reduction in Acute Coronary Syndrome; TROPICAL-ACS, Testing Responsiveness to Platelet Inhibition on Chronic Antiplatelet Treatment for Acute Coronary Syndromes; UFH, Un-Fractionated Heparin; VKA, Vitamin K Antagonist.

## References

[1] Neumann FJ, Sousa-Uva M, Ahlsson A, Alfonso F, Banning AP, Benedetto U (2019). 2018 ESC/EACTS Guidelines on myocardial revascularization [published correction appears in European Heart Journal. 2019; 40: 3096]. *European Heart Journal*.

[2] Angiolillo DJ, Galli M, Collet JP, Kastrati A, O’Donoghue ML (2022). Antiplatelet therapy after percutaneous coronary intervention. *EuroIntervention*.

[3] Simonsson M, Wallentin L, Alfredsson J, Erlinge D, Hellström Ängerud K, Hofmann R (2020). Temporal trends in bleeding events in acute myocardial infarction: insights from the SWEDEHEART registry. *European Heart Journal*.

[4] Valgimigli M, Costa F, Lokhnygina Y, Clare RM, Wallentin L, Moliterno DJ (2017). Trade-off of myocardial infarction vs. bleeding types on mortality after acute coronary syndrome: lessons from the Thrombin Receptor Antagonist for Clinical Event Reduction in Acute Coronary Syndrome (TRACER) randomized trial. *European Heart Journal*.

[5] Capodanno D, Bhatt DL, Gibson CM, James S, Kimura T, Mehran R (2022). Bleeding avoidance strategies in percutaneous coronary intervention. *Nature Reviews Cardiology*.

[6] Buccheri S, Capodanno D, James S, Angiolillo DJ (2019). Bleeding after antiplatelet therapy for the treatment of acute coronary syndromes: a review of the evidence and evolving paradigms. *Expert Opinion on Drug Safety*.

[7] Rao SV, O’Grady K, Pieper KS, Granger CB, Newby LK, Mahaffey KW (2006). A comparison of the clinical impact of bleeding measured by two different classifications among patients with acute coronary syndromes. *Journal of the American College of Cardiology*.

[8] Mehran R, Rao SV, Bhatt DL, Gibson CM, Caixeta A, Eikelboom J (2011). Standardized bleeding definitions for cardiovascular clinical trials: a consensus report from the Bleeding Academic Research Consortium. *Circulation*.

[9] Chesebro JH, Knatterud G, Roberts R, Borer J, Cohen LS, Dalen J (1987). Thrombolysis in Myocardial Infarction (TIMI) Trial, Phase i: a comparison between intravenous tissue plasminogen activator and intravenous streptokinase. Clinical findings through hospital discharge. *Circulation*.

[10] GUSTO investigators (1993). An international randomized trial comparing four thrombolytic strategies for acute myocardial infarction. *The New England Journal of Medicine*.

[11] Schulman S, Kearon C (2005). Definition of major bleeding in clinical investigations of antihemostatic medicinal products in non-surgical patients. *Journal of Thrombosis and Haemostasis*.

[12] Franco L, Becattini C, Beyer‐Westendorf J, Vanni S, Nitti C, Re R (2020). Definition of major bleeding: Prognostic classification. *Journal of Thrombosis and Haemostasis*.

[13] Navarese EP, Andreotti F, Schulze V, Kołodziejczak M, Buffon A, Brouwer M (2015). Optimal duration of dual antiplatelet therapy after percutaneous coronary intervention with drug eluting stents: meta-analysis of randomised controlled trials. *British Medical Journal*.

[14] van Rein N, Heide-Jørgensen U, Lijfering WM, Dekkers OM, Sørensen HT, Cannegieter SC (2019). Major Bleeding Rates in Atrial Fibrillation Patients on Single, Dual, or Triple Antithrombotic Therapy. *Circulation*.

[15] Franchi F, Angiolillo DJ (2015). Novel antiplatelet agents in acute coronary syndrome. *Nature Reviews Cardiology*.

[16] Lopes RD, Hong H, Harskamp RE, Bhatt DL, Mehran R, Cannon CP (2020). Optimal Antithrombotic Regimens for Patients with Atrial Fibrillation Undergoing Percutaneous Coronary Intervention: An Updated Network Meta-analysis. *JAMA Cardiology*.

[17] Thibert MJ, Fordyce CB, Cairns JA, Turgeon RD, Mackay M, Lee T (2021). Access-Site vs Non-Access-Site Major Bleeding and in-Hospital Outcomes among STEMI Patients Receiving Primary PCI. *CJC Open*.

[18] Caldeira D, Rodrigues FB, Barra M, Santos AT, de Abreu D, Gonçalves N (2015). Non-vitamin K antagonist oral anticoagulants and major bleeding-related fatality in patients with atrial fibrillation and venous thromboembolism: a systematic review and meta-analysis. *Heart*.

[19] Costa F, Valgimigli M, Steg PG, Bhatt DL, Hohnloser SH, Ten Berg JM (2022). Antithrombotic therapy according to baseline bleeding risk in patients with atrial fibrillation undergoing percutaneous coronary intervention: applying the PRECISE-DAPT score in re-DUAL PCI. *European Heart Journal - Cardiovascular Pharmacotherapy*.

[20] Chhatriwalla AK, Amin AP, Kennedy KF, House JA, Cohen DJ, Rao SV (2013). Association between Bleeding Events and in-hospital Mortality after Percutaneous Coronary Intervention. *JAMA*.

[21] Généreux P, Giustino G, Witzenbichler B, Weisz G, Stuckey TD, Rinaldi MJ (2015). Incidence, Predictors, and Impact of Post-Discharge Bleeding after Percutaneous Coronary Intervention. *Journal of the American College of Cardiology*.

[22] Piccolo R, Oliva A, Avvedimento M, Franzone A, Windecker S, Valgimigli M (2021). Mortality after bleeding versus myocardial infarction in coronary artery disease: a systematic review and meta-analysis. *EuroIntervention*.

[23] Mehran R, Baber U, Steg PG, Ariti C, Weisz G, Witzenbichler B (2013). Cessation of dual antiplatelet treatment and cardiac events after percutaneous coronary intervention (PARIS): 2 year results from a prospective observational study. *The Lancet*.

[24] Borissoff JI, Spronk HM, ten Cate H (2011). The hemostatic system as a modulator of atherosclerosis. *The New England Journal of Medicine*.

[25] Rao SV (2004). Relationship of Blood Transfusion and Clinical Outcomes in Patients with Acute Coronary Syndromes. *The Journal of the American Medical Association*.

[26] Subherwal S, Bach RG, Chen AY, Gage BF, Rao SV, Newby LK (2009). Baseline Risk of Major Bleeding in Non–ST–Segment–Elevation Myocardial Infarction: the CRUSADE (Can Rapid risk stratification of Unstable angina patients Suppress ADverse outcomes with Early implementation of the ACC/AHA Guidelines) Bleeding Score. *Circulation*.

[27] Collet JP, Thiele H, Barbato E, Barthélémy O, Bauersachs J, Bhatt DL (2021). 2020 ESC Guidelines for the management of acute coronary syndromes in patients presenting without persistent ST-segment elevation. *European Heart Journal*.

[28] Costa F, van Klaveren D, James S, Heg D, Räber L, Feres F (2017). Derivation and validation of the predicting bleeding complications in patients undergoing stent implantation and subsequent dual antiplatelet therapy (PRECISE-DAPT) score: a pooled analysis of individual-patient datasets from clinical trials. *The Lancet*.

[29] Valgimigli M, Bueno H, Byrne RA, Collet JP, Costa F, Jeppsson A (2018). 2017 ESC focused update on dual antiplatelet therapy in coronary artery disease developed in collaboration with EACTS: The Task Force for dual antiplatelet therapy in coronary artery disease of the European Society of Cardiology (ESC) and of the European Association for Cardio-Thoracic Surgery (EACTS). *European Heart Journal*.

[30] Urban P, Mehran R, Colleran R, Angiolillo DJ, Byrne RA, Capodanno D (2019). Defining High Bleeding Risk in Patients Undergoing Percutaneous Coronary Intervention. *Circulation*.

[31] Kereiakes DJ, Yeh RW, Massaro JM, Cutlip DE, Steg PG, Wiviott SD (2016). DAPT Score Utility for Risk Prediction in Patients with or without Previous Myocardial Infarction. *Journal of the American College of Cardiology*.

[32] Knuuti J, Wijns W, Saraste A, Capodanno D, Barbato E, Funck-Brentano C (2020). 2019 ESC Guidelines for the diagnosis and management of chronic coronary syndromes [published correction appears in European Heart Journal. 2020; 41: 4242]. *European Heart Journal*.

[33] Ibanez B, James S, Agewall S, Antunes MJ, Bucciarelli-Ducci C, Bueno H (2018). 2017 ESC Guidelines for the management of acute myocardial infarction in patients presenting with ST-segment elevation: The Task Force for the management of acute myocardial infarction in patients presenting with ST-segment elevation of the European Society of Cardiology (ESC). *European Heart Journal*.

[34] Ueki Y, Bär S, Losdat S, Otsuka T, Zanchin C, Zanchin T (2020). Validation of the Academic Research Consortium for High Bleeding Risk (ARC-HBR) criteria in patients undergoing percutaneous coronary intervention and comparison with contemporary bleeding risk scores. *EuroIntervention*.

[35] Kawashima H, Gao C, Takahashi K, Tomaniak M, Ono M, Hara H (2020). Comparative Assessment of Predictive Performance of PRECISE-DAPT, CRUSADE, and ACUITY Scores in Risk Stratifying 30-Day Bleeding Events. *Thrombosis and Haemostasis*.

[36] Choi SY, Kim MH, Lee KM, Ko YG, Yoon CH, Jo MK (2021). Comparison of Performance between ARC-HBR Criteria and PRECISE-DAPT Score in Patients Undergoing Percutaneous Coronary Intervention. *Journal of Clinical Medicine*.

[37] Kayani WT, Khan MR, Deshotels MR, Jneid H (2020). Challenges and Controversies in the Management of ACS in Elderly Patients. *Current Cardiology Reports*.

[38] Gall E, Lafont A, Varenne O, Dumas F, Cariou A, Picard F (2021). Balancing thrombosis and bleeding after out-of-hospital cardiac arrest related to acute coronary syndrome: a literature review. *Archives of Cardiovascular Diseases*.

[39] Chiarito M, Kini A, Roumeliotis A, Cao D, Power D, Sartori S (2021). Prevalence and Impact of High Bleeding Risk in Patients Undergoing Left Main Artery Disease PCI. *JACC: Cardiovascular Interventions*.

[40] Galli M, Porto I, Andreotti F, D’Amario D, Vergallo R, Della Bona R (2019). Early anticoagulation in the current management of NSTE-ACS: Evidence, guidelines, practice and perspectives. *International Journal of Cardiology*.

[41] Karlsson S, Andell P, Mohammad MA, Koul S, Olivecrona GK, James SK (2019). Editor’s Choice- Heparin pre-treatment in patients with ST-segment elevation myocardial infarction and the risk of intracoronary thrombus and total vessel occlusion. Insights from the TASTE trial. *European Heart Journal: Acute Cardiovascular Care*.

[42] Anderson JAM, Hirsh J, Yusuf S, Johnston M, Afzal R, Mehta SR (2010). Comparison of the anticoagulant intensities of fondaparinux and enoxaparin in the organization to assess strategies in acute ischemic syndromes (OASIS)-5 trial. *Journal of Thrombosis and Haemostasis*.

[43] Yusuf S, Mehta SR, Chrolavicius S, Afzal R, Pogue J, Granger CB (2006). Effects of fondaparinux on mortality and reinfarction in patients with acute ST-segment elevation myocardial infarction: the OASIS-6 randomized trial. *The Journal of the American Medical Association*.

[44] Galli M, Andreotti F, D’Amario D, Vergallo R, Vescovo GM, Giraldi L (2020). Antithrombotic therapy in the early phase of non-ST-elevation acute coronary syndromes: a systematic review and meta-analysis. *European Heart Journal - Cardiovascular Pharmacotherapy*.

[45] Ferguson JJ, Califf RM, Antman EM, Cohen M, Grines CL, Goodman S (2004). Enoxaparin vs unfractionated heparin in high-risk patients with non-ST-segment elevation acute coronary syndromes managed with an intended early invasive strategy: primary results of the SYNERGY randomized trial. *The Journal of the American Medical Association*.

[46] Capodanno D, Angiolillo DJ (2016). Reviewing the controversy surrounding pre-treatment with P2Y12 inhibitors in acute coronary syndrome patients. *Expert Review of Cardiovascular Therapy*.

[47] Généreux P, Stone GW, Harrington RA, Gibson CM, Steg PG, Brener SJ (2014). Impact of intraprocedural stent thrombosis during percutaneous coronary intervention: insights from the CHAMPION PHOENIX Trial (Clinical Trial Comparing Cangrelor to Clopidogrel Standard of Care Therapy in Subjects Who Require Percutaneous Coronary Intervention). *Journal of the American College of Cardiology*.

[48] Cavender MA, Bhatt DL, Stone GW, White HD, Steg PG, Gibson CM (2016). Consistent Reduction in Periprocedural Myocardial Infarction with Cangrelor as Assessed by Multiple Definitions: Findings From CHAMPION PHOENIX (Cangrelor Versus Standard Therapy to Achieve Optimal Management of Platelet Inhibition). *Circulation*.

[49] Dawson LP, Chen D, Dagan M, Bloom J, Taylor A, Duffy SJ (2021). Assessment of Pretreatment with Oral P2Y12 Inhibitors and Cardiovascular and Bleeding Outcomes in Patients with Non-ST Elevation Acute Coronary Syndromes: A Systematic Review and Meta-analysis. *JAMA Network Open*.

[50] Capranzano P, Angiolillo DJ (2021). Tackling the gap in platelet inhibition with oral antiplatelet agents in high-risk patients undergoing percutaneous coronary intervention. *Expert Review of Cardiovascular Therapy*.

[51] Sibbing D, Kastrati A, Berger PB (2016). Pre-treatment with P2Y12 inhibitors in ACS patients: who, when, why, and which agent. *European Heart Journal*.

[52] Montalescot G, van ‘t Hof AW, Lapostolle F, Silvain J, Lassen JF, Bolognese L (2014). Prehospital ticagrelor in ST-segment elevation myocardial infarction. *The New England Journal of Medicine*.

[53] Capodanno D, Milluzzo RP, Angiolillo DJ (2019). Intravenous antiplatelet therapies (glycoprotein IIb/IIIa receptor inhibitors and cangrelor) in percutaneous coronary intervention: from pharmacology to indications for clinical use. *Therapeutic Advances in Cardiovascular Disease*.

[54] Galli M, Migliaro S, Rodolico D, DI Stefano G, Piccinni C, Restivo A (2021). Intracoronary bolus of glycoprotein IIb/IIIa inhibitor as bridging or adjunctive strategy to oral P2Y12 inhibitor load in the modern setting of STEMI. *Minerva Cardiology and Angiology*.

[55] Ortega-Paz L, Galli M, Capodanno D, Brugaletta S, Angiolillo DJ (2022). The Role of Antiplatelet Therapy in Patients With MINOCA. *Frontiers in Cardiovascular Medicine*.

[56] Galli M, Angiolillo DJ (2021). Non-opioid analgesics in patients undergoing percutaneous coronary intervention: hype or hope. *European Heart Journal*.

[57] De Luca L, Leonardi S, Smecca IM, Formigli D, Lucci D, Gonzini L (2015). Contemporary antithrombotic strategies in patients with acute coronary syndromes managed without revascularization: insights from the EYESHOT study. *European Heart Journal - Cardiovascular Pharmacotherapy*.

[58] Montalescot G, Bolognese L, Dudek D, Goldstein P, Hamm C, Tanguay J (2013). Pretreatment with Prasugrel in Non–ST–Segment Elevation Acute Coronary Syndromes. *New England Journal of Medicine*.

[59] Schüpke S, Neumann FJ, Menichelli M, Mayer K, Bernlochner I, Wöhrle J (2019). Ticagrelor or Prasugrel in Patients with Acute Coronary Syndromes. *The New England Journal of Medicine*.

[60] Tarantini G, Mojoli M, Varbella F, Caporale R, Rigattieri S, Andò G (2020). Timing of Oral P2Y_⁢12 Inhibitor Administration in Patients With Non-ST-Segment Elevation Acute Coronary Syndrome. *Journal of the American College of Cardiology*.

[61] Rubboli A, Di Pasquale G (2015). Anti-platelet pre-treatment for atrial fibrillation patients on warfarin referred for coronary angiography/stenting because of non-ST-elevation acute coronary syndrome: an alternative proposal. *International Journal of Cardiology*.

[62] Jolly SS, Yusuf S, Cairns J, Niemelä K, Xavier D, Widimsky P (2011). Radial versus femoral access for coronary angiography and intervention in patients with acute coronary syndromes (RIVAL): a randomised, parallel group, multicentre trial. *The Lancet*.

[63] Valgimigli M, Frigoli E, Leonardi S, Vranckx P, Rothenbühler M, Tebaldi M (2018). Radial versus femoral access and bivalirudin versus unfractionated heparin in invasively managed patients with acute coronary syndrome (MATRIX): final 1-year results of a multicentre, randomised controlled trial. *The Lancet*.

[64] Romagnoli E, Biondi-Zoccai G, Sciahbasi A, Politi L, Rigattieri S, Pendenza G (2012). Radial versus femoral randomized investigation in ST-segment elevation acute coronary syndrome: the RIFLE-STEACS (Radial Versus Femoral Randomized Investigation in ST-Elevation Acute Coronary Syndrome) study. *Journal of the American College of Cardiology*.

[65] Mignatti A, Friedmann P, Slovut DP (2018). Targeting the safe zone: a quality improvement project to reduce vascular access complications. *Catheterization and Cardiovascular Interventions*.

[66] Rao SV, Stone GW (2016). Arterial access and arteriotomy site closure devices. *Nature Reviews Cardiology*.

[67] Finn AV, Nakazawa G, Joner M, Kolodgie FD, Mont EK, Gold HK (2007). Vascular Responses to Drug Eluting Stents. *Arteriosclerosis, Thrombosis, and Vascular Biology*.

[68] Palmerini T, Biondi-Zoccai G, Della Riva D, Mariani A, Genereux P, Branzi A (2013). Stent Thrombosis with Drug-Eluting Stents: is the paradigm shifting. *Journal of the American College of Cardiology*.

[69] Urban P, Abizaid A, Chevalier B, Greene S, Meredith I, Morice M (2013). Rationale and design of the LEADERS FREE trial: a randomized double-blind comparison of the BioFreedom drug-coated stent vs the Gazelle bare metal stent in patients at high bleeding risk using a short (1 month) course of dual antiplatelet therapy. *American Heart Journal*.

[70] Varenne O, Cook S, Sideris G, Kedev S, Cuisset T, Carrié D (2018). Drug-eluting stents in elderly patients with coronary artery disease (SENIOR): a randomised single-blind trial. *The Lancet*.

[71] Windecker S, Latib A, Kedhi E, Kirtane AJ, Kandzari DE, Mehran R (2020). Polymer-based or Polymer-free Stents in Patients at High Bleeding Risk. *New England Journal of Medicine*.

[72] Kozuma K, Kinoshita Y, Hioki H, Nanasato M, Ito Y, Yamaguchi J (2020). 1-Year Safety of 3-Month Dual Antiplatelet Therapy Followed by Aspirin or P2Y_⁢12 Receptor Inhibitor Monotherapy Using a Bioabsorbable Polymer Sirolimus-Eluting Stent. *Circulation Journal*.

[73] Mehran R, Cao D, Angiolillo DJ, Bangalore S, Bhatt DL, Ge J (2021). 3- or 1-Month DAPT in Patients at High Bleeding Risk Undergoing Everolimus-Eluting Stent Implantation. *JACC: Cardiovascular Interventions*.

[74] Urban P, Abizaid A, Banning A, Bartorelli AL, Baux AC, Džavík V (2011). Stent Thrombosis and Bleeding Complications after Implantation of Sirolimus-Eluting Coronary Stents in an Unselected Worldwide Population: a report from the e-SELECT (Multi-Center Post-Market Surveillance) registry. *Journal of the American College of Cardiology*.

[75] Burzotta F, Annone U, Paraggio L, D’Ascenzo F, Biondi-Zoccai G, Aurigemma C (2020). Clinical outcome after percutaneous coronary intervention with drug-eluting stent in bifurcation and nonbifurcation lesions: a meta-analysis of 23 981 patients. *Coronary Artery Disease*.

[76] Giustino G, Costa F (2019). Characterization of the Individual Patient Risk after Percutaneous Coronary Intervention: At the Crossroads of Bleeding and Thrombosis. *JACC: Cardiovascular Interventions*.

[77] Darmoch F, Alraies MC, Al‐Khadra Y, Moussa Pacha H, Pinto DS, Osborn EA (2020). Intravascular Ultrasound Imaging-Guided Versus Coronary Angiography-Guided Percutaneous Coronary Intervention: a Systematic Review and Meta‐Analysis. *Journal of the American Heart Association*.

[78] Vergallo R, De Maria GL, Annibali G, D’Amario D, Galli M, Porto I (2020). Use of intravascular ultrasound for the assessment and treatment of left main disease. *Giornale Italiano Di Cardiologia*.

[79] Ali ZA, Maehara A, Généreux P, Shlofmitz RA, Fabbiocchi F, Nazif TM (2016). Optical coherence tomography compared with intravascular ultrasound and with angiography to guide coronary stent implantation (ILUMIEN III: OPTIMIZE PCI): a randomised controlled trial. *The Lancet*.

[80] Colombo A, Hall P, Nakamura S, Almagor Y, Maiello L, Martini G (1995). Intracoronary stenting without anticoagulation accomplished with intravascular ultrasound guidance. *Circulation*.

[81] De Marzo V, D’amario D, Galli M, Vergallo R, Porto I (2018). High-risk percutaneous coronary intervention: how to define it today. *Minerva Cardioangiologica*.

[82] Grandin EW, Nunez JI, Willar B, Kennedy K, Rycus P, Tonna JE (2022). Mechanical Left Ventricular Unloading in Patients Undergoing Venoarterial Extracorporeal Membrane Oxygenation. *Journal of the American College of Cardiology*.

[83] Tehrani BN, Truesdell AG, Sherwood MW, Desai S, Tran HA, Epps KC (2019). Standardized Team-Based Care for Cardiogenic Shock [published correction appears in Journal of the American College of Cardiology. 2019; 74: 481]. *Journal of the American College of Cardiology*.

[84] Angiolillo DJ, Capodanno D (2015). Management of adjunctive antithrombotic therapy in STEMI patients treated with fibrinolysis undergoing rescue or delayed PCI. *Thrombosis and Haemostasis*.

[85] Gershlick AH, Stephens-Lloyd A, Hughes S, Abrams KR, Stevens SE, Uren NG (2005). Rescue Angioplasty after Failed Thrombolytic Therapy for Acute Myocardial Infarction. *New England Journal of Medicine*.

[86] Assessment of the Safety and Efficacy of a New Treatment Strategy with Percutaneous Coronary Intervention (ASSENT-4 PCI) investigators (2006). Primary versus tenecteplase-facilitated percutaneous coronary intervention in patients with ST-segment elevation acute myocardial infarction (ASSENT-4 PCI): randomised trial. *The Lancet*.

[87] Ellis SG, Armstrong P, Betriu A, Brodie B, Herrmann H, Montalescot G (2004). Facilitated percutaneous coronary intervention versus primary percutaneous coronary intervention: design and rationale of the Facilitated Intervention with Enhanced Reperfusion Speed to Stop Events (FINESSE) trial. *American Heart Journal*.

[88] Keeley EC, Boura JA, Grines CL (2006). Comparison of primary and facilitated percutaneous coronary interventions for ST-elevation myocardial infarction: quantitative review of randomised trials. *The Lancet*.

[89] Capodanno D, Huber K, Mehran R, Lip GYH, Faxon DP, Granger CB (2019). Management of Antithrombotic Therapy in Atrial Fibrillation Patients Undergoing PCI: JACC State-of-the-Art Review. *Journal of the American College of Cardiology*.

[90] Steffel J, Collins R, Antz M, Cornu P, Desteghe L, Haeusler KG (2021). 2021 European Heart Rhythm Association Practical Guide on the Use of Non-Vitamin K Antagonist Oral Anticoagulants in Patients with Atrial Fibrillation]. *Europace*.

[91] Galli M, Franchi F, Rollini F, Angiolillo DJ (2021). Role of platelet function and genetic testing in patients undergoing percutaneous coronary intervention. *Trends in Cardiovascular Medicine*.

[92] Sibbing D, Aradi D, Alexopoulos D, ten Berg J, Bhatt DL, Bonello L (2019). Updated Expert Consensus Statement on Platelet Function and Genetic Testing for Guiding P2Y12 Receptor Inhibitor Treatment in Percutaneous Coronary Intervention. *JACC: Cardiovascular Interventions*.

[93] Hahn JY, Song YB, Oh JH, Cho DK, Lee JB, Doh JH (2018). 6-month versus 12-month or longer dual antiplatelet therapy after percutaneous coronary intervention in patients with acute coronary syndrome (SMART-DATE): a randomised, open-label, non-inferiority trial. *The Lancet*.

[94] Valgimigli M, Frigoli E, Heg D, Tijssen J, Jüni P, Vranckx P (2021). Dual Antiplatelet Therapy after PCI in Patients at High Bleeding Risk. *New England Journal of Medicine*.

[95] Baber U, Zafar MU, Dangas G, Escolar G, Angiolillo DJ, Sharma SK (2020). Ticagrelor With or Without Aspirin After PCI: The TWILIGHT Platelet Substudy. *Journal of the American College of Cardiology*.

[96] Armstrong PC, Leadbeater PD, Chan MV, Kirkby NS, Jakubowski JA, Mitchell JA (2011). In the presence of strong P2Y12 receptor blockade, aspirin provides little additional inhibition of platelet aggregation. *Journal of Thrombosis and Haemostasis*.

[97] Galli M, Capodanno D, Andreotti F, Crea F, Angiolillo DJ (2021). Safety and efficacy of P2Y_12_ inhibitor monotherapy in patients undergoing percutaneous coronary interventions. *Expert Opinion on Drug Safety*.

[98] Natsuaki M, Morimoto T, Yamamoto E, Shiomi H, Furukawa Y, Abe M (2016). One-year outcome of a prospective trial stopping dual antiplatelet therapy at 3 months after everolimus-eluting cobalt-chromium stent implantation: ShortT and OPtimal duration of Dual AntiPlatelet Therapy after everolimus-eluting cobalt-chromium stent (STOPDAPT) trial. *Cardiovascular Intervention and Therapeutics*.

[99] Hahn JY, Song YB, Oh JH, Chun WJ, Park YH, Jang WJ (2019). Effect of P2Y12 Inhibitor Monotherapy vs Dual Antiplatelet Therapy on Cardiovascular Events in Patients Undergoing Percutaneous Coronary Intervention: The SMART-CHOICE Randomized Clinical Trial. *The Journal of the American Medical Association*.

[100] Watanabe H, Morimoto T, Natsuaki M, Yamamoto K, Obayashi Y, Ogita M (2022). Comparison of Clopidogrel Monotherapy After 1 to 2 Months of Dual Antiplatelet Therapy With 12 Months of Dual Antiplatelet Therapy in Patients With Acute Coronary Syndrome: The STOPDAPT-2 ACS Randomized Clinical Trial. *JAMA Cardiology*.

[101] O’Donoghue ML, Murphy SA, Sabatine MS (2020). The Safety and Efficacy of Aspirin Discontinuation on a Background of a P2Y12 Inhibitor in Patients After Percutaneous Coronary Intervention: A Systematic Review and Meta-Analysis. *Circulation*.

[102] Kim B, Hong S, Cho Y, Yun KH, Kim YH, Suh Y (2020). Effect of Ticagrelor Monotherapy vs Ticagrelor with Aspirin on Major Bleeding and Cardiovascular Events in Patients with Acute Coronary Syndrome: The TICO Randomized Clinical Trial. *The Journal of the American Medical Association*.

[103] Kogame N, Guimarães PO, Modolo R, De Martino F, Tinoco J, Ribeiro EE (2020). Aspirin-Free Prasugrel Monotherapy Following Coronary Artery Stenting in Patients With Stable CAD: The ASET Pilot Study. *JACC: Cardiovascular Interventions*.

[104] Galli M, Franchi F, Rollini F, Cavallari LH, Capodanno D, Crea F (2021). Genetic testing in patients undergoing percutaneous coronary intervention: rationale, evidence and practical recommendations. *Expert Review of Clinical Pharmacology*.

[105] Wallentin L, Becker RC, Budaj A, Cannon CP, Emanuelsson H, Held C (2009). Ticagrelor versus clopidogrel in patients with acute coronary syndromes. *New England Journal of Medicine*.

[106] Wiviott SD, Braunwald E, McCabe CH, Montalescot G, Ruzyllo W, Gottlieb S (2007). Prasugrel versus clopidogrel in patients with acute coronary syndromes. *The New England Journal of Medicine*.

[107] Aradi D, Kirtane A, Bonello L, Gurbel PA, Tantry US, Huber K (2015). Bleeding and stent thrombosis on P2Y12-inhibitors: collaborative analysis on the role of platelet reactivity for risk stratification after percutaneous coronary intervention. *European Heart Journal*.

[108] Pereira NL, Rihal C, Lennon R, Marcus G, Shrivastava S, Bell MR (2021). Effect of CYP2C19 Genotype on Ischemic Outcomes during Oral P2Y12 Inhibitor Therapy: A Meta-Analysis. *JACC: Cardiovascular Interventions*.

[109] Cayla G, Cuisset T, Silvain J, Leclercq F, Manzo-Silberman S, Saint-Etienne C (2016). Platelet function monitoring to adjust antiplatelet therapy in elderly patients stented for an acute coronary syndrome (ANTARCTIC): an open-label, blinded-endpoint, randomised controlled superiority trial. *The Lancet*.

[110] Sibbing D, Aradi D, Jacobshagen C, Gross L, Trenk D, Geisler T (2017). Guided de-escalation of antiplatelet treatment in patients with acute coronary syndrome undergoing percutaneous coronary intervention (TROPICAL-ACS): a randomised, open-label, multicentre trial. *The Lancet*.

[111] Claassens DMF, Vos GJA, Bergmeijer TO, Hermanides RS, van ‘t Hof AWJ, van der Harst P (2019). A Genotype-Guided Strategy for Oral P2Y_⁢12 Inhibitors in Primary PCI. *The New England Journal of Medicine*.

[112] Galli M, Benenati S, Capodanno D, Franchi F, Rollini F, D’Amario D (2021). Guided versus standard antiplatelet therapy in patients undergoing percutaneous coronary intervention: a systematic review and meta-analysis. *The Lancet*.

[113] Galli M, Benenati S, Franchi F, Rollini F, Capodanno D, Biondi-Zoccai G (2022). Comparative effects of guided vs. potent P2Y12 inhibitor therapy in acute coronary syndrome: a network meta-analysis of 61 898 patients from 15 randomized trials. *European Heart Journal*.

[114] Cuisset T, Deharo P, Quilici J, Johnson TW, Deffarges S, Bassez C (2017). Benefit of switching dual antiplatelet therapy after acute coronary syndrome: the TOPIC (timing of platelet inhibition after acute coronary syndrome) randomized study. *European Heart Journal*.

[115] Kim H, Kang J, Hwang D, Han J, Yang H, Kang H (2020). Prasugrel-based de-escalation of dual antiplatelet therapy after percutaneous coronary intervention in patients with acute coronary syndrome (HOST-REDUCE-POLYTECH-ACS): an open-label, multicentre, non-inferiority randomised trial. *The Lancet*.

[116] Park M, Kim CJ, Kim M, Choo E, Hwang B, Park C (2021). A prospective, multicentre, randomised, open-label trial to compare the efficacy and safety of clopidogrel versus ticagrelor in stabilised patients with acute myocardial infarction after percutaneous coronary intervention: rationale and design of the TALOS-AMI trial. *EuroIntervention*.

[117] Kralev S, Schneider K, Lang S, Süselbeck T, Borggrefe M (2011). Incidence and severity of coronary artery disease in patients with atrial fibrillation undergoing first-time coronary angiography. *PLoS ONE*.

[118] Hindricks G, Potpara T, Dagres N, Arbelo E, Bax JJ, Blomström-Lundqvist C (2021). 2020 ESC Guidelines for the diagnosis and management of atrial fibrillation developed in collaboration with the European Association for Cardio-Thoracic Surgery (EACTS): The Task Force for the diagnosis and management of atrial fibrillation of the European Society of Cardiology (ESC) Developed with the special contribution of the European Heart Rhythm Association (EHRA) of the ESC [published correction appears in European Heart Journal. 2021 Feb 1;42(5):507] [published correction appears in European Heart Journal. 2021; 42: 546–547] [published correction appears in European Heart Journal2021; 42: 4194]. *European Heart Journal*.

[119] Angiolillo DJ, Bhatt DL, Cannon CP, Eikelboom JW, Gibson CM, Goodman SG (2021). Antithrombotic Therapy in Patients With Atrial Fibrillation Treated With Oral Anticoagulation Undergoing Percutaneous Coronary Intervention: A North American Perspective: 2021 Update. *Circulation*.

[120] Frisullo G, Profice P, Brunetti V, Scala I, Bellavia S, Broccolini A (2020). Prospective Observational Study of Safety of Early Treatment with Edoxaban in Patients with Ischemic Stroke and Atrial Fibrillation (SATES Study). *Brain Sciences*.

[121] Gibson CM, Mehran R, Bode C, Halperin J, Verheugt F, Wildgoose P (2015). An open-label, randomized, controlled, multicenter study exploring two treatment strategies of rivaroxaban and a dose-adjusted oral vitamin K antagonist treatment strategy in subjects with atrial fibrillation who undergo percutaneous coronary intervention (PIONEER AF-PCI). *American Heart Journal*.

[122] Lopes RD, Vora AN, Liaw D, Granger CB, Darius H, Goodman SG (2018). An open-Label, 2 × 2 factorial, randomized controlled trial to evaluate the safety of apixaban vs. vitamin K antagonist and aspirin vs. placebo in patients with atrial fibrillation and acute coronary syndrome and/or percutaneous coronary intervention: Rationale and design of the AUGUSTUS trial. *American Heart Journal*.

[123] Cannon CP, Bhatt DL, Oldgren J, Lip GYH, Ellis SG, Kimura T (2017). Dual Antithrombotic Therapy with Dabigatran after PCI in Atrial Fibrillation. *New England Journal of Medicine*.

[124] Vranckx P, Valgimigli M, Eckardt L, Tijssen J, Lewalter T, Gargiulo G (2019). Edoxaban-based versus vitamin K antagonist-based antithrombotic regimen after successful coronary stenting in patients with atrial fibrillation (ENTRUST-AF PCI): a randomised, open-label, phase 3b trial. *The Lancet*.

[125] Galli M, Andreotti F, Porto I, Crea F (2020). Intracranial haemorrhages vs. stent thromboses with direct oral anticoagulant plus single antiplatelet agent or triple antithrombotic therapy: a meta-analysis of randomized trials in atrial fibrillation and percutaneous coronary intervention/acute coronary syndrome patients. *Europace*.

[126] Gargiulo G, Goette A, Tijssen J, Eckardt L, Lewalter T, Vranckx P (2019). Safety and efficacy outcomes of double vs. triple antithrombotic therapy in patients with atrial fibrillation following percutaneous coronary intervention: a systematic review and meta-analysis of non-vitamin K antagonist oral anticoagulant-based randomized clinical trials. *European Heart Journal*.

[127] Galli M, Andreotti F, D’Amario D, Vergallo R, Montone RA, Niccoli G (2020). Randomised trials and meta-analyses of double vs triple antithrombotic therapy for atrial fibrillation-ACS/PCI: A critical appraisal. *International Journal of Cardiology: Heart & Vasculature*.

[128] De Caterina R, Agewall S, Andreotti F, Angiolillo DJ, Bhatt DL, Byrne RA (2022). Great Debate: Triple antithrombotic therapy in patients with atrial fibrillation undergoing coronary stenting should be limited to 1 week. *European Heart Journal*.

[129] Agewall S, Cattaneo M, Collet JP, Andreotti F, Lip GY, Verheugt FW (2013). Expert position paper on the use of proton pump inhibitors in patients with cardiovascular disease and antithrombotic therapy. *European Heart Journal*.

[130] Lai KC, Lam SK, Chu KM, Wong BC, Hui WM, Hu WH (2002). Lansoprazole for the prevention of recurrences of ulcer complications from long-term low-dose aspirin use. *The New England Journal of Medicine*.

[131] Bhatt DL, Cryer BL, Contant CF, Cohen M, Lanas A, Schnitzer TJ (2010). Clopidogrel with or without Omeprazole in Coronary Artery Disease. *New England Journal of Medicine*.

[132] O’Donoghue ML, Braunwald E, Antman EM, Murphy SA, Bates ER, Rozenman Y (2009). Pharmacodynamic effect and clinical efficacy of clopidogrel and prasugrel with or without a proton-pump inhibitor: an analysis of two randomised trials. *The Lancet*.

[133] Ndrepepa G, Groha P, Lahmann AL, Lohaus R, Cassese S, Schulz-Schüpke S (2016). Increased bleeding risk during percutaneous coronary interventions by arterial hypertension. *Catheterization and Cardiovascular Interventions*.

[134] Olsen AS, McGettigan P, Gerds TA, Fosbøl EL, Olesen JB, Sindet-Pedersen C (2020). Risk of gastrointestinal bleeding associated with oral anticoagulation and non-steroidal anti-inflammatory drugs in patients with atrial fibrillation: a nationwide study. *European Heart Journal - Cardiovascular Pharmacotherapy*.

[135] Frontera JA, Lewin JJ, Rabinstein AA, Aisiku IP, Alexandrov AW, Cook AM (2016). Guideline for Reversal of Antithrombotics in Intracranial Hemorrhage: A Statement for Healthcare Professionals from the Neurocritical Care Society and Society of Critical Care Medicine. *Neurocritical Care*.

[136] Baharoglu MI, Cordonnier C, Salman RA, de Gans K, Koopman MM, Brand A (2016). Platelet transfusion versus standard care after acute stroke due to spontaneous cerebral haemorrhage associated with antiplatelet therapy (PATCH): a randomised, open-label, phase 3 trial. *The Lancet*.

[137] Li X, Sun Z, Zhao W, Zhang J, Chen J, Li Y (2013). Effect of acetylsalicylic acid usage and platelet transfusion on postoperative hemorrhage and activities of daily living in patients with acute intracerebral hemorrhage. *Journal of Neurosurgery*.

[138] Desborough MJR, Oakland KA, Landoni G, Crivellari M, Doree C, Estcourt LJ (2017). Desmopressin for treatment of platelet dysfunction and reversal of antiplatelet agents: a systematic review and meta‐analysis of randomized controlled trials. *Journal of Thrombosis and Haemostasis*.

[139] Zafar MU, Smith DA, Baber U, Sartori S, Chen K, Lam DW (2017). Impact of Timing on the Functional Recovery Achieved With Platelet Supplementation After Treatment With Ticagrelor. *Circulation: Cardiovascular interventions*.

[140] Bhatt DL, Pollack CV, Mazer CD, Angiolillo DJ, Steg G, James SK (2021). Bentracimab for Ticagrelor Reversal in Patients Undergoing Urgent Surgery. *NEJM Evidence*.

[141] Ageno W, Garcia D, Aguilar MI, Douketis J, Finazzi G, Imberti D (2009). Prevention and treatment of bleeding complications in patients receiving vitamin K antagonists, part 2: Treatment. *American Journal of Hematology*.

[142] Ageno W, Gallus AS, Wittkowsky A, Crowther M, Hylek EM, Palareti G (2012). Oral anticoagulant therapy: Antithrombotic Therapy and Prevention of Thrombosis, 9th ed: American College of Chest Physicians Evidence-Based Clinical Practice Guidelines. *Chest*.

[143] Levy JH, Douketis J, Weitz JI (2018). Reversal agents for non-vitamin K antagonist oral anticoagulants. *Nature Reviews Cardiology*.

[144] Siegal DM, Curnutte JT, Connolly SJ, Lu G, Conley PB, Wiens BL (2015). Andexanet alfa for the Reversal of Factor Xa Inhibitor Activity. *New England Journal of Medicine*.

[145] Angiolillo DJ, Capodanno D, Danchin N, Simon T, Bergmeijer TO, Ten Berg JM (2020). Derivation, Validation, and Prognostic Utility of a Prediction Rule for Nonresponse to Clopidogrel: The ABCD-GENE Score. *JACC: Cardiovascular Interventions*.

[146] De Caterina R, D’Ugo E, Libby P (2016). Inflammation and thrombosis – testing the hypothesis with anti- inflammatory drug trials. *Thrombosis and Haemostasis*.

[147] Galli M, Capodanno D, Benenati S, D’Amario D, Crea F, Andreotti F (2021). Efficacy and safety of dual pathway inhibition in patients with cardiovascular disease: a systematic review and Meta-analysis. *European Heart Journal: Cardiovascular Pharmacotherapy*.

[148] Galli M, Franchi F, Rollini F, Been L, Jaoude PA, Rivas A (2022). Pharmacodynamic Profiles of Dual-Pathway Inhibition with or without Clopidogrel versus Dual Antiplatelet Therapy in Patients with Atherosclerotic Disease. *Thrombosis and Haemostasis*.

[149] Galli M, Franchi F, Rollini F, Been L, Jaoude PA, Rivas A (2022). Platelet P2Y12 inhibiting therapy in adjunct to vascular dose of rivaroxaban or aspirin: A pharmacodynamic study of dual pathway inhibition versus dual antiplatelet therapy. *European Heart Journal: Cardiovascular Pharmacotherapy*.

[150] Hsu C, Hutt E, Bloomfield DM, Gailani D, Weitz JI (2021). Factor XI Inhibition to Uncouple Thrombosis from Hemostasis: JACC Review Topic of the Week. *Journal of the American College of Cardiology*.

[151] Heitmeier S, Visser M, Tersteegen A, Dietze‐Torres J, Glunz J, Gerdes C (2022). Pharmacological profile of asundexian, a novel, orally bioavailable inhibitor of factor XIa. *Journal of Thrombosis and Haemostasis*.

[152] Büller HR, Bethune C, Bhanot S, Gailani D, Monia BP, Raskob GE (2015). Factor XI antisense oligonucleotide for prevention of venous thrombosis. *The New England Journal of Medicine*.

[153] Gailani D (2022). Making anticoagulation safer. *The Lancet*.

[154] Cryer B, Mahaffey KW (2014). Gastrointestinal ulcers, role of aspirin, and clinical outcomes: pathobiology, diagnosis, and treatment. *Journal of Multidisciplinary Healthcare*.

[155] Angiolillo DJ, Bhatt DL, Lanza F, Cryer B, Dong JF, Jeske W (2019). Pharmacokinetic/pharmacodynamic assessment of a novel, pharmaceutical lipid-aspirin complex: results of a randomized, crossover, bioequivalence study. *Journal of Thrombosis and Thrombolysis*.

[156] Ansell J, Laulicht BE, Bakhru SH, Burnett A, Jiang X, Chen L (2021). Ciraparantag, an anticoagulant reversal drug: mechanism of action, pharmacokinetics, and reversal of anticoagulants. *Blood*.

[157] Ansell J, Bakhru S, Laulicht BE, Tracey G, Villano S, Freedman D (2022). Ciraparantag reverses the anticoagulant activity of apixaban and rivaroxaban in healthy elderly subjects. *European Heart Journal*.

[158] Smetana KS, Counts J, Sodhi A, May CC (2022). Review of Target-Specific Anticoagulation Reversal Agents. *Critical Care Nursing Quarterly*.

